# Engineered crRNA Drives RPA‐T7‐CRISPR/Cas14a Cascade for Ultrasensitive Detection of ctDNA PIK3CA H1047R

**DOI:** 10.1002/advs.202507126

**Published:** 2025-08-30

**Authors:** Yuanyuan Yu, Mengru Jin, Weiguang Yuan, Yajie Gong, Siwei Li, Xuquan Qin, Jianxun Hou, Jialin Liu, Siyu Liu, Hui Li, Yijun Chu, Yingjie Wang, Youxue Zhang, Fang Fang, Wenhui Hao, Yuling Gu, Qinchen Fan, Jing Lin, Da Pang, Xianyu Zhang

**Affiliations:** ^1^ Department of Breast Surgery Harbin Medical University Cancer Hospital Harbin Heilongjiang 150086 China; ^2^ Institute of Cancer Prevention and Treatment Harbin Medical University Heilongjiang Academy of Medical Sciences Harbin Heilongjiang 150086 China; ^3^ Shanghai Naturethink Life & Scientific Co. Ltd Shanghai 201809 China

**Keywords:** CRISPR/Cas14a, ctDNA detection, diagnostics, PIK3CA H1047R, RPA

## Abstract

The early detection of circulating tumor DNA (ctDNA) at mutant allele frequencies below 0.1% remains a critical challenge, significantly impeding therapeutic decision‐making. To address this limitation, TIDE‐Cas14a—an innovative CRISPR/Cas14a‐based duplex detection system is developed that integrates recombinase polymerase amplification (RPA) with T7 exonuclease‐mediated strand displacement. By strategically engineering crRNAs with synthetic mismatches, the platform achieves single‐nucleotide resolution, enabling specific discrimination of the PIK3CA H1047R (c.3140A>G) variant from other mutant subtypes and wild‐type sequences at a detection limit of 0.01% with attomolar sensitivity. The system leverages T7 exonuclease's 5′→3′ digestion to convert RPA amplicons into single‐stranded targets, thereby activating Cas14a without requiring thermal cycling. Furthermore, clinical validation using 32 breast cancer patient samples demonstrated that TIDE‐Cas14a achieves 100% sensitivity and specificity, comparable to droplet digital PCR. When deployed on a low‐cost digital microfluidic chip, the assay completes ctDNA profiling within 60 min at 37 °C, effectively bridging the gap between complex laboratory testing and point‐of‐care diagnostics. The work repurposes the CRISPR/Cas system's inherent specificity constraints as a precision oncology tool, establishing a scalable platform for early cancer detection and therapeutic monitoring.

## Graphical abstract

The TIDE‐Cas14a system, an integrated RPA‐T7‐CRISPR/Cas14a cascade platform incorporating engineered crRNA mismatches, enables ultrasensitive detection of the PIK3CA H1047R mutation in breast cancer circulating tumor DNA, with a limit of detection as low as 0.01%, surpassing the sensitivity of droplet digital PCR. When coupled with a digital microfluidic chip, this platform offers a rapid and cost‐effective solution for early cancer diagnosis and minimal residual disease monitoring, demonstrating strong clinical potential in precision oncology applications.

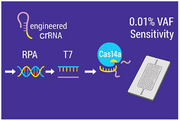

## 1 Introduction

Currently, genetic testing has become a cornerstone of early cancer diagnosis and treatment, dynamic monitoring, and prognostic evaluation, with precision oncology profoundly reshaping cancer diagnosis and management paradigms.^[^ ^1^ ^]^ Traditional tissue biopsies face limitations due to invasive procedural risks, restricted sample accessibility, and tumor heterogeneity‐induced detection biases, hindering their utility for dynamic monitoring and personalized therapy.^[^ ^2^ ^]^ In this context, liquid biopsy technologies targeting circulating tumor DNA (ctDNA) have emerged as revolutionary tools for early detection, offering non‐invasiveness, real‐time capability, and the ability to overcome tumor heterogeneity. However, a critical challenge for liquid biopsy lies in the extremely low proportion of ctDNA in the peripheral blood of early‐stage cancer patients. Conventional detection methods fail to identify low‐variant allele frequency (VAF) subclonal mutations (<0.1%) responsible for drug resistance, underscoring the urgent need for ultrasensitive ctDNA monitoring technologies.^[^ ^3^, ^4^, ^5^, ^6^, ^7^ ^]^

Highly sensitive technologies such as droplet digital PCR (ddPCR) and next‐generation sequencing (NGS) have improved the detection of low‐abundance mutant DNA in clinical samples.^[^ ^8^, ^9^, ^10^, ^11^ ^]^ NGS enables comprehensive mutational profiling in complex genomic backgrounds, facilitating the detection and quantification of diverse low‐frequency variants, including single nucleotide variants, insertions/deletions, copy number variations, and structural variations. However, its reliance on intricate library preparation workflows, high‐throughput sequencing platforms, and sophisticated bioinformatics tools for data analysis results in prolonged operational procedures and elevated technical barriers, often necessitating specialized personnel. Additionally, the high costs associated with equipment procurement and maintenance may render NGS less cost‐effective for targeted mutation screening. Furthermore, specific strategies such as barcoding and target enrichment are required to achieve a standard NGS analytical sensitivity of 1%.^[^ ^12^, ^13^, ^14^ ^]^ In contrast, ddPCR can identify and quantify ultra‐rare mutations with VAFs as low as 0.1% amidst a high background of wild‐type cell‐free DNA (cfDNA), and enables absolute quantification of nucleic acids. Similar to NGS, ddPCR depends on thermal cyclers, complex instrumentation, extended processing times, and specialized expertise, along with expensive laboratory infrastructure, thereby limiting its broad applicability in point‐of‐care testing (POCT).^[^ ^15^, ^16^ ^]^

The advent of isothermal amplification technologies has revolutionized nucleic acid detection by eliminating dependence on thermal cyclers, yet significant limitations persist in clinical implementation. Techniques such as loop‐mediated isothermal amplification and rolling circle amplification, which avoid thermal cycling, often suffer from complex primer design and nonspecific amplification, thereby compromising both sensitivity and specificity.^[^ ^17^, ^18^, ^19^, ^20^, ^21^ ^]^ In contrast, recombinase polymerase amplification (RPA) exhibits unique advantages for liquid biopsy applications. Specifically, it requires only two primers for amplification, achieves high sensitivity without complex equipment, and is particularly well‐suited for POCT. However, nonspecific primer annealing in blood‐derived samples generates false‐positive amplicons, reducing the signal‐to‐noise ratio and undermining test accuracy.

Clustered regularly interspaced short palindromic repeats (CRISPR) and CRISPR‐associated proteins (collectively referred to as the CRISPR/Cas system, including Cas12a, Cas13a, and Cas14a) have revolutionized in recent years due to their precise targeting capability, high sensitivity, and exceptional specificity, and have been widely applied in nucleic acid molecular diagnostics through their cis‐cleavage and trans‐cleavage activities.^[^ ^22^, ^23^, ^24^, ^25^ ^]^ The Cas14a protein is the smallest known class 2 CRISPR RNA‐guided enzymes, ≈400‐700 amino acids, can target and cleave any single‐stranded DNA (ssDNA) with random sequences under the guidance of single‐guide RNA (sgRNA) without requiring a protospacer adjacent motif (PAM) sequence, while also activating its trans‐cleavage activity against ssDNA. Compared with CRISPR/Cas12a, it exhibits higher specificity and more stable recognition and cleavage performance.^[^ ^26^, ^27^, ^28^ ^]^ Current research data demonstrates that the sensitivity of CRISPR‐based direct detection currently reaches only the picomolar level, which is insufficient for identifying low‐abundance genetic mutations in the blood of early‐stage cancer patients.^[^ ^29^, ^30^, ^31^ ^]^ Therefore, CRISPR‐based diagnostic technologies must employ target sequence pre‐amplification to enhance detection performance. Given that the optimal temperature for Cas14a protease activity aligns with that required for RPA, we have achieved a 0.01% limit of detection (LOD) by integrating RPA with CRISPR/Cas14a technology. This innovative system features a dual optimization mechanism. First, isothermal amplification enables exponential enrichment of target nucleic acids, significantly improving detection signal sensitivity. Second, the sequence‐specific recognition and cleavage capability of the CRISPR/Cas14a system effectively eliminates background interference caused by nonspecific byproducts during RPA amplification, thereby substantially enhancing detection specificity.^[^ ^32^ ^]^

Breast cancer (BC) remains the most prevalent malignancy among women worldwide, with over 2.3 million new cases annually and 685000 deaths.^[^ ^33^, ^34^, ^35^, ^36^ ^]^ While early diagnosis has substantially improved clinical outcomes, evidenced by a 5‐year survival rate surpassing 90% for localized disease, the prognosis plummets to ≈32% once distant metastasis occurs, underscoring the critical need for timely detection.^[^ ^37^, ^38^, ^39^, ^40^, ^41^ ^]^ Molecularly, dysregulation of the PI3K‐AKT‐mTOR signaling axis, a central regulator of cellular proliferation and survival, is implicated in 70% of BC through genetic alterations such as mutations or amplifications.^[^ ^42^, ^43^ ^]^ Among these, the PIK3CA p.H1047R (c.3140A>G) mutation in the kinase domain (exon 20), the most frequent genetic variant detectable in 30–40% of BC cases, constitutively activates PI3Kα by destabilizing the autoinhibitory interaction between the p110α catalytic and p85 regulatory subunits.^[^ ^44^, ^45^, ^46^, ^47^, ^48^ ^]^ Clinically, this oncogenic driver induces estrogen receptor‐independent survival pathways, thereby driving both primary and acquired resistance to endocrine therapies in hormone receptor‐positive BC.^[^ ^49^ ^]^ Given the profound impact of PI3K pathway activation on therapeutic response and disease progression, developing ultrasensitive detection methods for early‐stage malignancies emerges as a pivotal strategy to mitigate treatment resistance and improve long‐term survival.

Our study establishes a novel T7 exonuclease‐mediated strand‐conversion strategy combined with CRISPR/Cas14a for the specific detection of the breast cancer‐associated ctDNA PIK3CA H1047R mutation.^[^ ^26^, ^32^, ^50^, ^51^ ^]^ Unlike conventional approaches, our system utilizes the 5′→3′ double‐stranded DNA (dsDNA) digestion specificity of T7 exonuclease. During RPA, the enzyme selectively degrades the antisense strand with phosphorylated 5′ ends while preserving the sense strand through phosphorothioate (PT) modifications, enabling thermal cycle‐free ssDNA conversion for direct Cas14a recognition of RPA amplicons. Building on SHERLOCK findings that base mismatches enhance single‐nucleotide discrimination in CRISPR/Cas13a, we establish, for the first time, that this principle generalizes to CRISPR/Cas14a.^[^ ^52^ ^]^ By strategically introducing synthetic single‐nucleotide mismatches at defined positions within the CRISPR RNA (crRNA) sequence, we achieved highly specific detection of the PIK3CA H1047R mutation, enabling single‐nucleotide discrimination from wild‐type sequences. This high‐fidelity genotyping capability was further validated across clinically relevant cancer genes, including EGFR, BRAF and KRAS for key oncogenic mutations in lung and colorectal cancers. We term this integrated platform T7‐Integrated Duplex Enhancer CRISPR/Cas14a (TIDE‐Cas14a). To rigorously evaluate the absolute quantification capacity of our CRISPR/Cas14a‐based rapid mutation detection system, we performed method validation using tissue and paired plasma samples from BC patients. We used ddPCR as the gold standard reference while establishing a highly sensitive liquid biopsy detection platform through parallel implementation and comparative analysis with digital detection strategies.

## 2 Results and Discussion

### 2.1 Workflow of the TIDE‐Cas14a System

Single nucleotide polymorphisms (SNPs) represent critical biomarkers in cancer diagnostics.^[^ ^53^, ^54^ ^]^ However, their detection presents substantial challenges due to the extremely low VAF in ctDNA from clinical samples. To address the need for highly sensitive and specific SNP mutation detection, we developed the TIDE‐Cas14a system.^[^ ^26^, ^52^ ^]^ This innovative one‐pot detection technology integrates RPA‐driven target amplification, T7 exonuclease‐mediated strand‐specific digestion, and CRISPR/Cas14a cleavage specificity to achieve rapid and precise SNP detection. The sgRNA of CRISPR/Cas14a complex consists of trans‐activating CRISPR RNA (tracrRNA) and crRNA, with crRNA sequences being specifically engineered in this study. The “Duplex Enhancer” mechanism combines RPA amplification efficiency with engineered crRNA design to achieve single‐nucleotide resolution.

**Figure 1:**
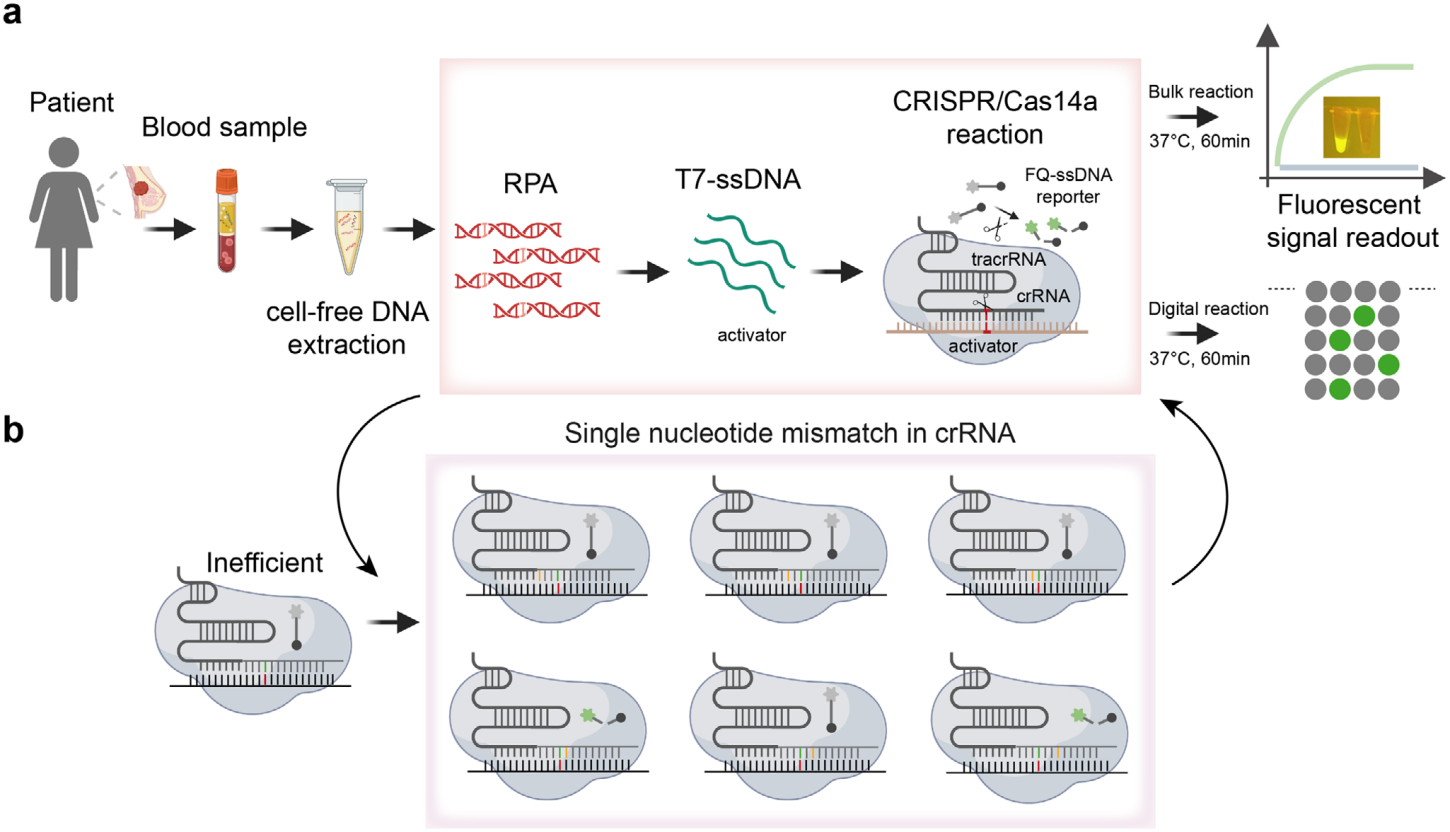
Workflow of the TIDE‐Cas14a system. a) Patient‐derived ctDNA is extracted from plasma samples and detected via a one‐pot RPA‐T7‐CRISPR/Cas14a reaction. The reaction mixture contains RPA reagents, RPA primers, T7 exonuclease, Cas14a protein, sgRNA, and a FAM‐quenched single‐stranded DNA (FQ‐ssDNA) fluorescent reporter. During the isothermal reaction at 37 °C, mutant and wild‐type alleles are rapidly amplified. T7‐exo selectively retains the phosphorothioate (PT)‐modified sense strand, while the Cas14a/sgRNA complex specifically recognizes target sequences in the sample, triggering trans‐cleavage of the FQ‐ssDNA reporter by Cas14a to release fluorescent signals. Fluorescence signals are visualized using a portable blue‐light transilluminator or quantitatively analyzed via a high‐throughput digital microfluidic chip, with the entire detection process completed within 1 h. b) Engineered crRNA, designed by introducing base substitutions at distinct positions, enhances the recognition specificity and cleavage efficiency of the Cas14a/sgRNA complex toward target sequences. Created in BioRender. Yu, Y. (2025) https://BioRender.com/n04v877.

In summary, the ctDNA extracted from plasma is directly added into a single‐tube reaction system containing the RPA mixture, T7 exonuclease, Cas14a protein, single‐stranded DNA fluorescent probes, and engineered crRNA. The target sequences are first amplified by RPA, which generates dsDNA amplicons even from trace mutant alleles. Subsequently, T7 exonuclease selectively degrades the phosphorylated 5′‐end antisense strand of RPA amplicons while leaving the sense strand protected by PT modification for Cas14a recognition. Crucially, we enhanced the stability of the Cas14a/crRNA‐ssDNA ternary complex and its target recognition specificity by introducing residue substitutions at non‐mutation sites of the crRNA. Despite over 99.9% sequence similarity between wild‐type DNA and mutant sequences, the single‐nucleotide mismatch at SNP sites prevents Cas14a activation, thereby ensuring stringent system specificity. Upon recognition of target mutations in samples, the Cas14a protein becomes activated, triggering its collateral cleavage activity that cuts both the target ssDNA and the ssDNA reporter probe (FAM‐ssDNA‐BHQ1), releasing real‐time fluorescent signals detectable by portable fluorometers at a constant 37 °C. The one‐step RPA‐T7‐CRISPR/Cas14a reaction can be completed within 60 min (**Figure** **1**). Furthermore, we integrated the optimized RPA‐T7‐CRISPR system into our laboratory‐developed microfluidic chip, which discretizes the reaction mixture containing nucleic acids into tens of thousands of nanoliter‐scale droplets through 100000 microwells ‐ each droplet either containing or lacking the target gene, enabling digital detection. The entire reaction process completes within 60 min at 37 °C, and fluorescence microscopy detection of the chip's fluorescent signals demonstrates significantly enhanced sensitivity.

### 2.2 Design and Selection of crRNAs for the Specific Sensing of PIK3CA H1047R Mutation

**Figure 2:**
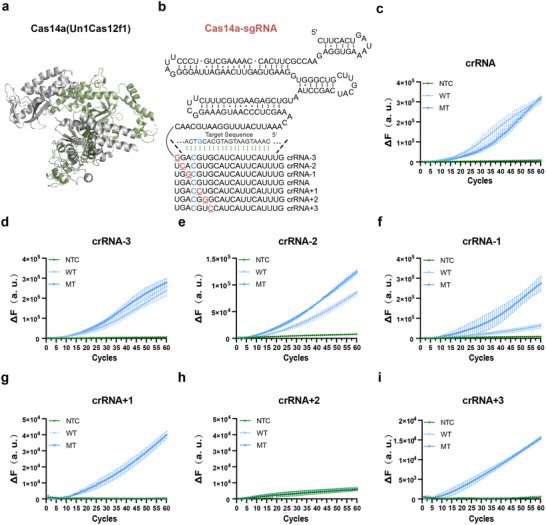
Engineering of crRNA modifications to improve signal‐to‐noise ratio. a) 3D structure of Cas14a protein (PDB ID: 7c7l). b) Schematic for targeting PIK3CA H1047R mutation SNPs in the crRNA are highlighted in blue, and synthetic mismatches are highlighted in red. Created in https://BioRender.com c‐i) Screening specific crRNA by inserting additional mismatche at different locations for a one‐pot RPA‐T7‐CRISPR/Cas14a detection system. Data in (c‐i) represent the means ± standard deviation (s.d.) (n = 3). NTC, no‐template control. WT, wild template. MT, mutant template. Source data are provided as a Source Data file.

To validate the feasibility of our proposed detection method, we employed the TIDE‐Cas14a system to detect the PIK3CA H1047R mutation hotspot, which is renowned for its high mutation frequency in BC patients. Previous studies demonstrated that the Cas14a (Un1Cas12f1) (PDB ID: 7c71) protein derived from uncultivated archaea has been experimentally characterized to cleave ssDNA in a PAM‐independent manner while demonstrating stringent specificity for single‐nucleotide polymorphisms SNPs (**Figure** **2**a).^[^ ^26^, ^55^ ^]^ Furthermore, Cas14a's target recognition triggers the collateral cleavage of nonspecific ssDNA molecules, enabling high‐fidelity SNP detection. This fidelity originates from Cas14a's hypersensitivity to base mismatches within its seed region, characterized by exceptionally low mismatch tolerance, which permits precise discrimination of single‐nucleotide variations.

We initially designed a crRNA with perfect complementarity to the PIK3CA H1047R target sequence (no base substitutions). While this unmodified crRNA induced significant fluorescence signal amplification compared to blank controls, it failed to distinguish mutant from wild‐type alleles (Figure 2b,c). Zhang Feng's team demonstrated that to increase the specificity of SHERLOCK, they introduced synthetic mismatches in the crRNA, which enabled LwCas13a to discriminate between targets that differ by a single‐base mismatch.^[^ ^52^ ^]^ Another study showed that introducing mismatches near the center of the ssDNA target strongly inhibited Cas14a activity, indicating that Cas14a requires complementarity within a seed region for recognition of ssDNA substrates. These findings suggest that CRISPR/Cas14a may employ a mechanism similar to that used by Cas13a enzymes targeting ssRNA.^[^ ^26^ ^]^ Based on these strategies, we systematically engineered crRNA sequences by introducing additional single‐base mismatches at specific positions within the target‐complementary crRNA region. This approach significantly improved target recognition specificity ‐ a strategy previously unreported for Cas14a or other Cas14 proteins. As illustrated in Figure 2b, we designed six crRNA variants incorporating random G/C substitutions at positions ‐3, ‐2, ‐1, +1, +2, and +3 relative to the SNP site. Each engineered crRNA was combined with Cas14a protein, ssDNA fluorescent probes, and 1 µL of ssDNA template. The reaction mixtures were incubated at 37 °C, with fluorescence signals quantified using an Applied Biosystems StepOnePlus Real‐Time PCR System. Surprisingly, results demonstrated that single‐nucleotide mismatches introduced at positions +1 or +3 relative to the SNP site enhanced discrimination between mutant and wild‐type alleles (Figure 2d–i). Since crRNA+1 exhibited a threefold higher signal‐to‐noise ratio compared to crRNA+3, we chose crRNA+1 for subsequent experiments.

**Figure 3:**
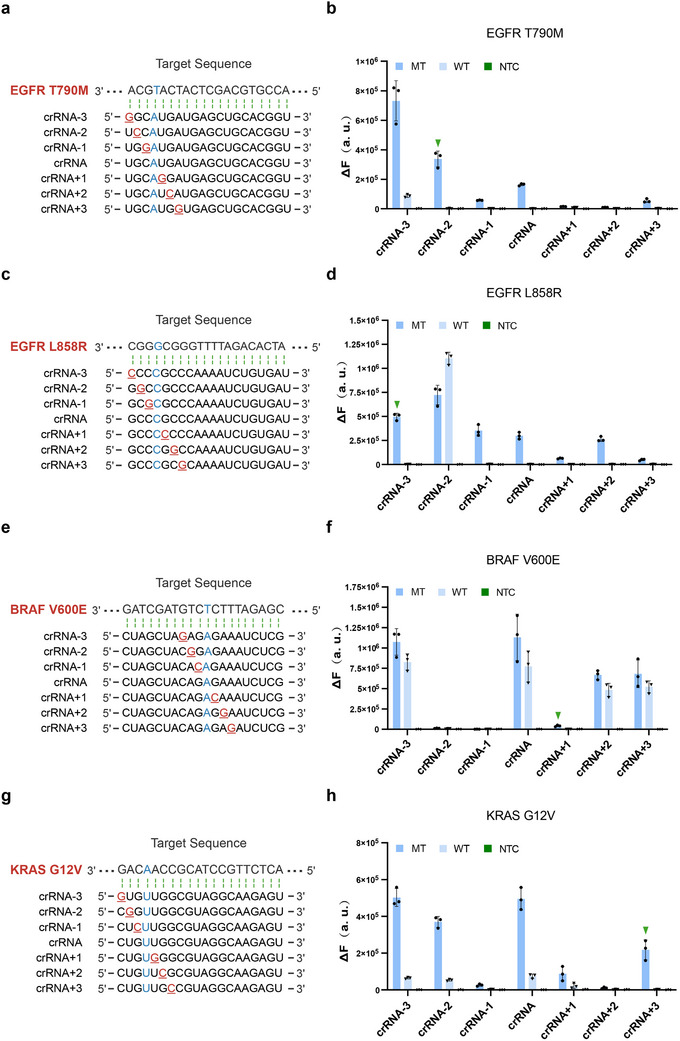
Engineered crRNA enables specific detection of clinically relevant oncogenic mutations. a, c, e, g) Schematic of target regions and crRNA variants for EGFR T790M, EGFR L858R, BRAF V600E, and KRAS G12V. Target SNPs are highlighted in blue, with engineered mismatches in the guide sequence (‐3 to +3 positions) shown in red. b, d, f, h) Detection specificity for EGFR T790M, EGFR L858R, BRAF V600E, and KRAS G12V mutation using mismatched crRNA designs. Data in (b, d, f, h) represent the means ± standard deviation (s.d.) (n = 3). NTC, no‐template control. WT, wild template. MT, mutant template. Source data are provided as a Source Data file. To enhance clarity, Figures 3b,d,f,h and S2 (Supporting Information) share the same source data.

To validate the broad applicability of this strategy, we systematically applied the crRNA mismatch engineering approach to additional clinically relevant mutations. As illustrated in **Figure** **3**, we employed identical crRNA design principles for key oncogenic mutations in lung and colorectal cancers, including EGFR T790M, EGFR L858R, BRAF V600E, and KRAS G12V, by introducing single‐nucleotide mismatches at positions ‐3 to +3 relative to each SNP site. The experimental data demonstrated robust discrimination between mutant and wild‐type templates across all tested mutations (Figure 3b,d,f,h; Figures S1 and S2, Supporting Information). Of particular significance, optimal detection specificity was achieved with crRNA‐2 for EGFR T790M, crRNA‐3 for EGFR L858R, crRNA+1 for BRAF V600E, and crRNA+3 for KRAS G12V. These findings collectively establish the general utility of our crRNA mismatch engineering platform for specific identification of diverse clinically relevant single‐nucleotide variants.

In addition, to further explore the underlying mechanism of this enhanced specificity, we performed molecular docking simulations to assess the impact of crRNA mismatches on the stability of the Cas14a/sgRNA/ssDNA ternary complex. For each of the five gene loci mentioned above, we carried out docking simulations using both the original crRNA and the selected best specificity crRNA. The docking results are shown in Figure S3 (Supporting Information).^[^ ^56^ ^]^ The docking scores of the ternary complexes containing mismatched crRNAs were all more negative than those with the original crRNA, suggesting that the Cas14a/sgRNA complexes formed with mismatched crRNAs exhibited reduced affinity toward the wild‐type ssDNA, compared to those formed with the original crRNA. Notably, for the wild‐type EGFR L858R gene, the docking scores showed no significant change. This is understandable, as both the original crRNA and the selected high‐specificity mismatched crRNA‐2 were able to clearly distinguish between the mutant and wild‐type EGFR L858R alleles (Figure 3d; Figure S2, Supporting Information).

### 2.3 Establishment and Optimization of the TIDE‐Cas14a System

**Figure 4:**
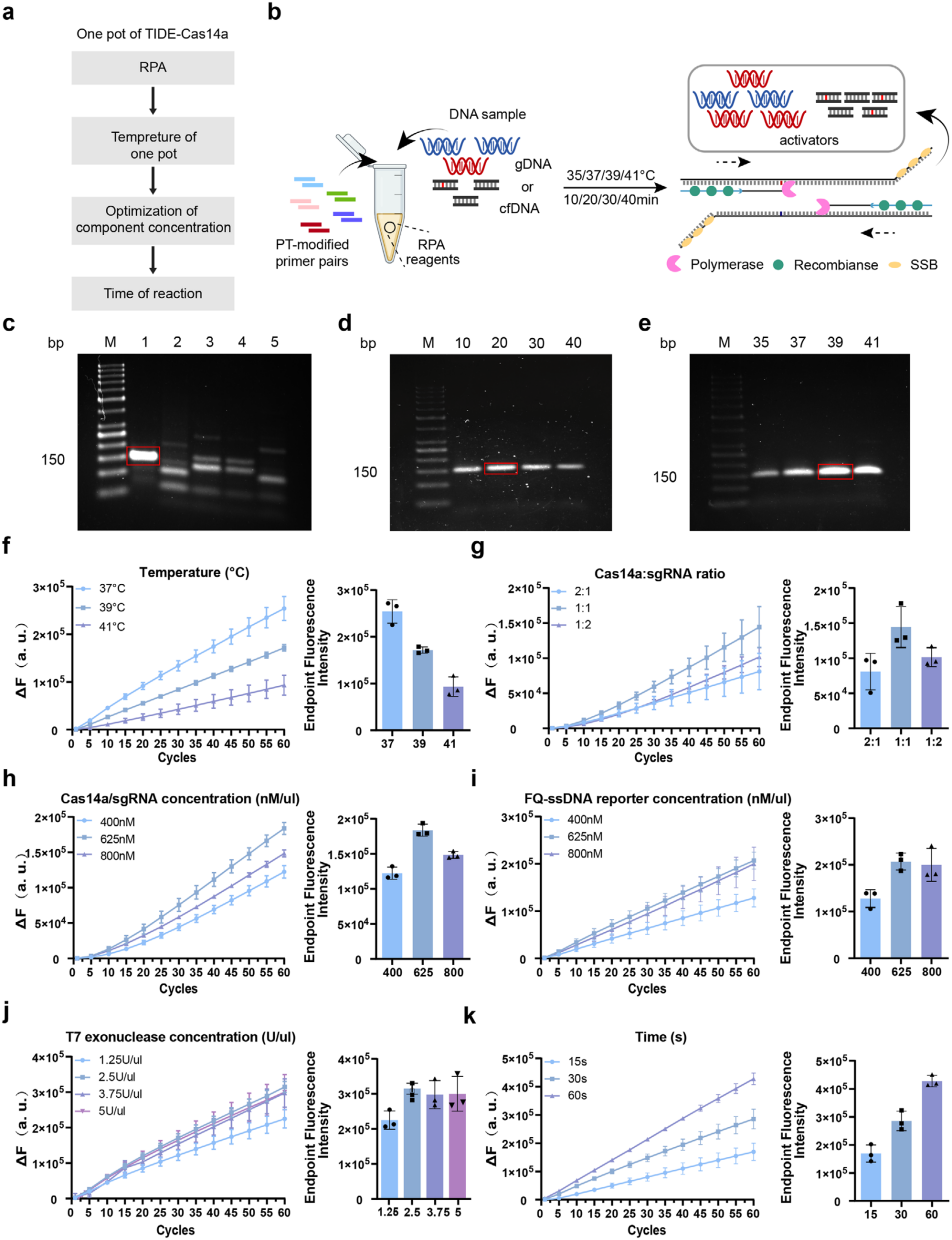
Establishment and optimization of the TIDE‐Cas14a system. a) Optimization process of the TIDE‐Cas14a system detection system. Reaction performed with 100 ng/µL PIK3CA H1047R target DNA(c)‐(k). b) Schematic of RPA amplification system optimization targeting the PIK3CA H1047R mutation site. c) Agarose gel electrophoresis result of RPA amplification with five primer pairs F1R1, F2R2, F2R3, F2R4, and F2R5. d, e) Agarose gel electrophoresis result of RPA reaction time and temperature optimization using primer pair F1R1. f) Temperature optimization for one‐pot TIDE‐Cas14a system in detecting PIK3CA H1047R mutation. g, h) Ratio and concentration optimization of the Cas14a and sgRNA. i) Concentration optimization of the FAM‐ssDNA‐BHQ1 reporter. j) Concentration optimization of the T7 exonuclease. k) Time optimization of the TIDE‐Cas14a system for detecting PIK3CA H1047R mutation. Data in (f‐k) represent the means ± standard deviation (s.d.) (n = 3). Source data are provided as a Source Data file.

Building upon the established detection conditions, we optimized the overall reaction performance for compatibility with our biosensor detection platform (**Figure** **4**a). As reaction temperature and duration critically influence RPA amplification efficiency, the optimization workflow for the RPA amplification system is illustrated in Figure 4b. First, we screened for optimal primer pairs using the target gene PIK3CA H1047R at an initial concentration of 100 ng/µL as the template, evaluating two forward primers (F1, F2) and five reverse primers (R1‐R5) (Table S1, Supporting Information). Following the manufacturer's recommendations, primer screening was conducted at 39 °C for 20 min. As shown in Figure 4c, the F1R1 primer pair successfully amplified the target gene without generating significant nonspecific amplification products or cross‐reactivity. Subsequent RPA amplifications with the F1R1 primer pair were performed under varying conditions, with reaction durations of 10, 20, 30, and 40 min, and temperatures of 35, 37, 39, and 41 °C. Results demonstrated maximal RPA amplification efficiency at 39 °C for 20 min, establishing these parameters as the optimal reaction conditions (Figure 4d,e).

Next, we attempted to combine the RPA isothermal amplification system with the T7 exonuclease and CRISPR/Cas14a detection systems to establish a one‐pot RPA‐T7‐CRISPR/Cas14a detection system. We initially validated the detection system using a two‐step approach, where 5 µL of RPA amplification products were added to the T7‐CRISPR/Cas14a system and reacted at 37 °C for 30 min. This resulted in significantly enhanced fluorescence signals with clear discrimination between mutant and wild‐type alleles, demonstrating the technical feasibility of combining CRISPR/Cas14 detection with RPA isothermal amplification (Figure S4, Supporting Information). Subsequently, to maximize the sensitivity of the detection system, we optimized the 20 µL one‐pot RPA‐T7‐CRISPR/Cas14a reaction system based on manufacturer recommendations and previous studies, as the concentration and ratio of reaction components significantly affect detection efficiency.^[^ ^23^, ^26^, ^57^, ^58^ ^]^

First, we studied different reaction temperatures (37, 39, and 42 °C), finding that 37 °C yielded the highest fluorescence signal (Figure 4f). Next, we optimized the ratio and concentration of Cas14a and sgRNA, determining that a 1:1 ratio at 625 nm each produced the strongest fluorescence signal (Figure 4g,h). Fluorescence intensity increased with higher concentrations of the FQ‐ssDNA probe. Based on both fluorescence intensity and signal‐to‐noise ratio, we selected 625 nM FQ‐ssDNA probe as the optimal condition (Figure 4i). When T7 exonuclease concentration reached 2.5 U µL^−1^, fluorescence intensity showed no significant difference, so we chose 2.5 U µL^−1^ for subsequent experiments (Figure 4j). Finally, we evaluated the effect of different reaction times on fluorescence intensity and, considering time costs, established 60 min as the incubation period without further optimization (Figure 4k).

In summary, we chose 480 nM RPA primers,^[^ ^18^ ^]^ 625 nM Cas14a, 625 nM sgRNA, 625 nM FQ‐ssDNA probe, and 2.5 U µL^−1^ T7 exonuclease as the optimal reaction parameters for subsequent experiments. To characterize the target‐activated cis‐cleavage of ssDNA and the non‐specific trans‐cleavage activity of CRISPR/Cas14a, we established control groups with different component omissions in the RPA‐T7‐CRISPR/Cas14a reaction system. After adding 1 µL of 1 ng µL^−1^ PIK3CA H1047R‐mut template at low concentration, the reaction mixture was incubated at 37 °C for 60 min in a real‐time PCR system with fluorescence signals collected every minute, followed by visual detection using a portable blue‐light transilluminator at 485 nm. Results showed that only when all components were present could the CRISPR/Cas14a system be activated by the target to cleave the FQ‐ssDNA probe and generate fluorescence (Figure S5, Supporting Information). These results confirm the feasibility of the target‐activated CRISPR/Cas14a signal amplification strategy.

### 2.4 Performance Analysis of TIDE‐Cas14a System for the Detection of PIK3CA H1047R Mutation

**Figure 5:**
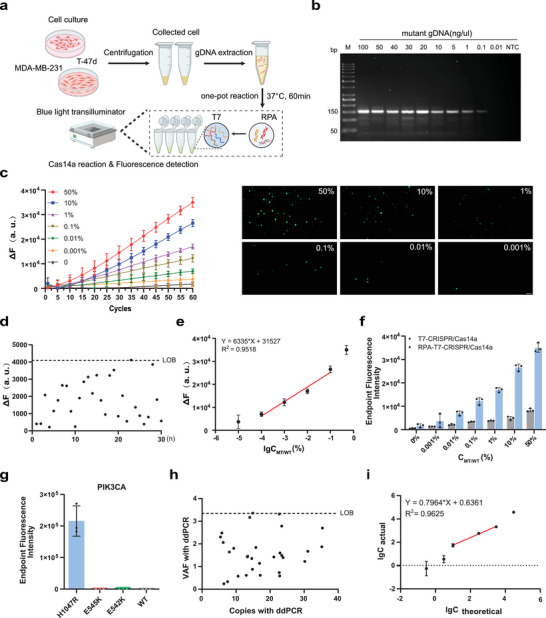
Analysis of the TIDE‐Cas14a system performance in detecting PIK3CA H1047R mutation in cell line genomic DNA. a) Schematic of TIDE‐Cas14a detection of PIK3CA H1047R mutation in cell lines. Created in https://BioRender.com b) Agarose gel electrophoresis of the RPA system for the sensitivity study of PIK3CA H1047R mutation detection. c) Real‐Time Fluorescence Curve of the gDNA‐based LOD assay for PIK3CA H1047R mutation detection. Representative endpoint fluorescence images of the digital microfluidic chip incubated at 37 °C were recorded to determine the LOD. Scale bars are 100 µm. d) LOB determination based on RPA‐T7‐CRISPR/Cas14a analysis of 30 healthy donor peripheral blood samples, with the 95th percentile threshold indicated (dashed line). e) Evaluation of the TIDE‐Cas14a's ability to detect different VAF. The linear relationship between the logarithm of the input VAF (X) and the fluorescence intensity (Y) measured by TIDE‐Cas14a is Y = 6335*X + 31527 (R^2^ = 0.9518). f) Comparison of endpoint fluorescence increments of RPA‐T7‐CRISPR/Cas14a and T7‐CRISPR/Cas14a. The target used in the T7‐CRISPR/Cas14a system is a synthetic fragment, in which the sense strand is modified with phosphorothioate (PT). g) The detection specificity of PIK3CA H1047R mutation and other mutant subtypes. h) LOB determination based on ddPCR analysis of 30 healthy donor peripheral blood samples, with the 95th percentile threshold indicated (dashed line). i) The linear relationship between input copies and ddPCR‐measured copies is Y = 0.7964*X + 0.6361 (R^2^ = 0.9625). Data in (c‐f) and (i) represent the means ±s.d. (n = 3). To enhance clarity, Figures 4c,e and S6 (Supporting Information) share the same source data. Source data are provided as a Source Data file.

To evaluate the quantitative capability of the TIDE‐Cas14a detection platform for nucleic acid analysis and its applicability in detecting SNPs from human samples, we employed gDNA extracted from BC cell lines to simulate physiological conditions, while comparing its performance with the absolute quantification capability of ddPCR (**Figure** **5**a). The PIK3CA H1047R mutant gDNA templates were subjected to tenfold serial dilution, with 1 µL of each dilution used for RPA amplification sensitivity testing. Agarose gel electrophoresis revealed faint but detectable bands at 152 bp even at the low template concentration of 0.1 ng µL^−1^, demonstrating that the RPA amplification sensitivity reached 0.1 ng µL^−1^ (≈30 copies µL^−1^) (Figure 5b).

For comprehensive assessment of the LOD of the TIDE‐Cas14a system, we prepared DNA templates with varying VAFs of 50%, 10%, 1%, 0.1%, 0.01%, and 0.001% by mixing PIK3CA H1047R mutant and wild‐type (MT/WT) gDNA at initial concentrations of 100 ng/µL. As shown in Figures 5c–e and S6 (Supporting Information), the fluorescence intensity response exhibited a progressive enhancement corresponding to increasing VAF ratios. Notably, an ideal linear relationship (R^2^ = 0.9518) was observed between ΔF and the logarithmic values of VAF ratios within the range of 0.01%‐10% MT/WT ratios. These results indicate that the TIDE‐Cas14a system achieved an LOD of 0.01%. Figure 5c further demonstrates that the proportion of positive spots on the microfluidic chip increased progressively with higher VAFs, with distinct differentiation between positive and negative spots under fluorescence microscopy. Detailed examination of fluorescence images revealed detectable signals even at 0.001% VAF, suggesting that the microfluidics‐based TIDE‐Cas14a system possesses single‐molecule detection capability. Comparative analysis confirmed that the detection performance of the TIDE‐Cas14a system significantly surpassed that of the standalone T7‐CRISPR/Cas14a assay (Figure 5f). We further validated the specificity of TIDE‐Cas14a. Figure 5g shows that it precisely identified the PIK3CA H1047R mutation subtype with no cross‐reactivity. This high specificity is critical for BC applications because clonal hematopoiesis and tumor heterogeneity can interfere with mutation detection in plasma samples.^[^ ^59^ ^]^

We subsequently compared the performance of TIDE‐Cas14a and ddPCR methodologies across varying VAF levels to evaluate the sensitivity of ddPCR in detecting PIK3CA H1047R mutations. The initial optimization of ddPCR assays involved primer specificity analysis and temperature gradient experiments, which established 63 °C as the optimal annealing temperature for PIK3CA H1047R mutation‐specific primers (Figure S7, Supporting Information). To establish baseline parameters, we analyzed cfDNA from the peripheral blood plasma of 30 healthy volunteers. The Shapiro‐Wilk test confirmed a right‐skewed distribution of background signals, prompting the use of non‐parametric methods for limit of blank (LOB) determination (Figure 5h). The calculated LOB was 35.3 copies/µL (VAF 3.33%), with all ddPCR results below this threshold reported as “not detected”. For direct comparison, by mixing PIK3CA H1047R mutant and wild‐type gDNA under identical DNA input amounts (100 ng/µl) and sample batch conditions, we prepared DNA templates with different VAF levels of 50%, 10%, 1%, 0.1%, 0.01%, and 0.001% (n = 4) to calculate the LOD of ddPCR (Figure S8, Supporting Information). The results showed that TIDE‐Cas14a consistently detected PIK3CA H1047R mutations at VAF levels as low as 0.01% through fluorescence threshold analysis, significantly outperforming the LOD of ddPCR (0.1%) (Figure 5i). This superior sensitivity highlights the substantial potential of TIDE‐Cas14a for detecting low‐abundance VAFs in liquid biopsy applications.

Reproducibility and repeatability are crucial parameters for the practical application of the TIDE‐Cas14a system. Figure S9 (Supporting Information) shows the fluorescence signal intensity for detecting the EGFR L858R mutation using crRNA‐3 under various conditions, including different dates (“Exp. 1”, “Exp. 2”, “Exp. 3”), different operators (“Operator A”, “Operator B”, “Operator C”), and different Cas14a protein reagent lots (“Lot #1”, “Lot #2”, “Lot #3”). As illustrated in the figure, the system demonstrates a high degree of consistency and reproducibility across different dates, operators, and reagent lots. These findings indicate that the TIDE‐Cas14a system possesses robustness and reliability, making it suitable for clinical diagnostic deployment.

### 2.5 Detection of PIK3CA H1047R Mutation in Clinical Samples Using the TIDE‐Cas14a System

To evaluate the detection capability of TIDE‐Cas14a for fragmented cfDNA, a key characteristic of plasma‐derived cfDNA (≈166 bp), we extracted cfDNA from the culture supernatants of T47D (PIK3CA H1047R mutant) and MDA‐MB‐231 (wild‐type control for PIK3CA H1047R) cells using a cell‐free DNA extraction kit. As shown in Figure S10 (Supporting Information), PT‐modified RPA primers demonstrated comparable enrichment efficiency for target sequences relative to conventional RPA primers. Importantly, TIDE‐Cas14a successfully distinguished the PIK3CA H1047R mutation in cfDNA derived from cell lines, highlighting its potential for mutation detection in liquid biopsy applications.^[^ ^60^, ^61^, ^62^, ^63^ ^]^

**Figure 6:**
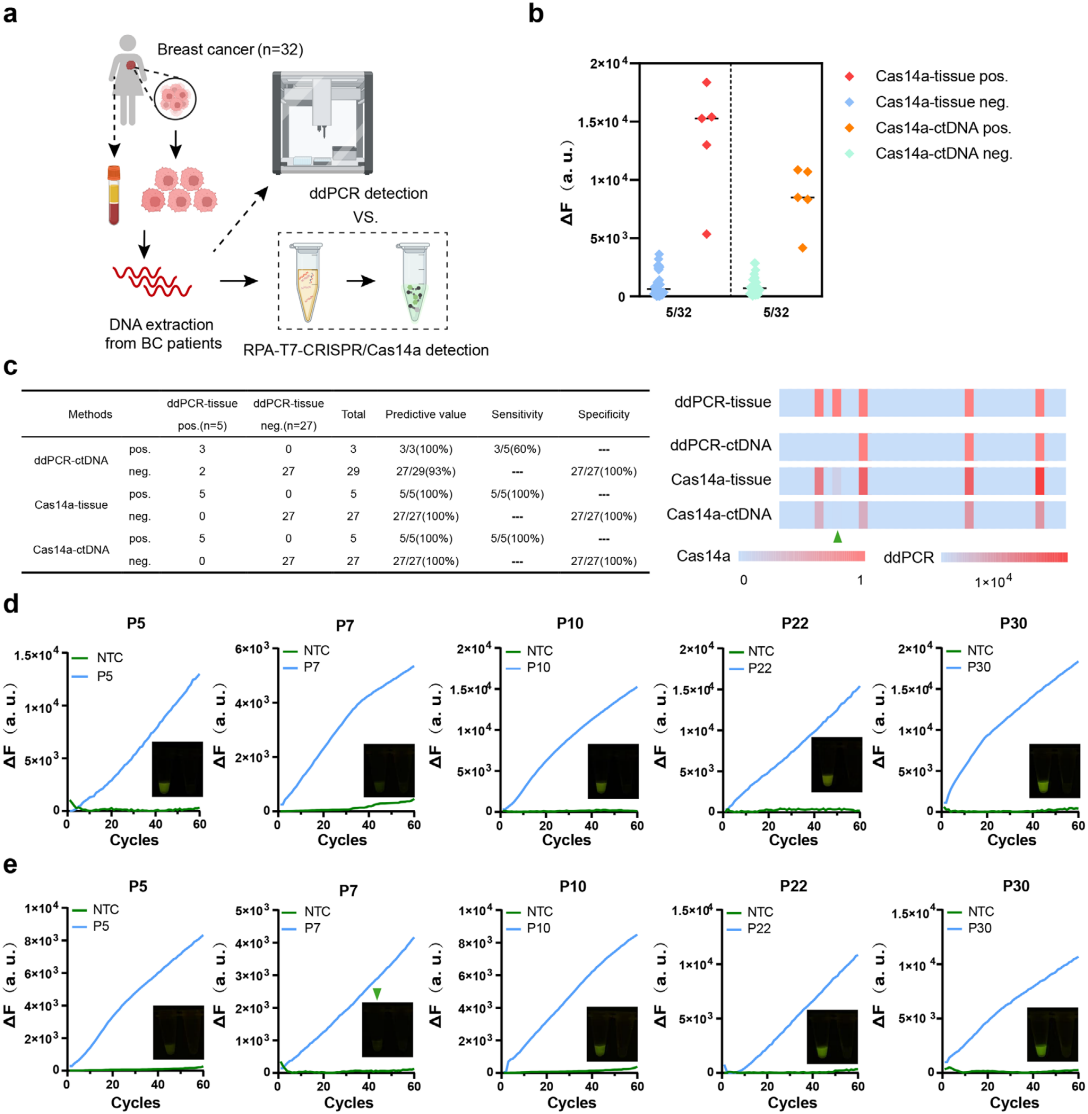
Detection of PIK3CA H1047R mutation in clinical samples using the TIDE‐Cas14a system and ddPCR. a) Schematic of PIK3CA H1047R detection in human BC tissue and paired plasma sample using TIDE‐Cas14a and ddPCR. Tumor tissue and paired plasma samples were collected from 32 BC patients to determine the sensitivity and specificity of both detection methods. Created in https://BioRender.com b) The detection of PIK3CA H1047R mutations in tissue and paired plasma samples using the TIDE‐Cas14a assay. c) Statistical table and heatmap of the sensitivity and specificity of the TIDE‐Cas14a method in detecting 32 BC patients compared with ddPCR and using tissue ddPCR results as a gold standard reference. d) Realtime fluorescence curves and visual detection results of positive clinical samples (P5, P7, P10, P22, P30) in tissue samples using the TIDE‐Cas14a method. e) Realtime fluorescence curves and visual detection results of positive clinical samples (P5, P7, P10, P22, P30) in plasma samples using the TIDE‐Cas14a method.

The detection performance of biosensors ultimately depends on their capability in clinical samples. To evaluate the detection sensitivity and specificity of TIDE‐Cas14a in clinical practice, we collected 32 tissue and paired plasma samples from BC patients at Harbin Medical University Cancer Hospital (Tables S3 and S4, Supporting Information). As shown in **Figure** **6**a, in this study we considered the ddPCR analysis results of PIK3CA H1047R mutations in BC tumor tissues as the gold standard reference. After DNA extraction from both tumor tissues and plasma, detection was performed using ddPCR and TIDE‐Cas14a methods separately. Figure 6b demonstrates that positive and negative samples could be clearly distinguished based on fluorescence values. Analysis of BC tissue gDNA showed high concordance between TIDE‐Cas14a and ddPCR detection methods, with TIDE‐Cas14a achieving 100% sensitivity (5/5) and 100% specificity (27/27) (Figure 6c).

Subsequently, we further compared the clinical performance of TIDE‐Cas14a and ddPCR methods in detecting PIK3CA H1047R mutations in ctDNA. The results showed complete consistency between Cas14a‐ctDNA and ddPCR‐tissue detection results (Figure S11a, Supporting Information). Figure 6d,e presents the detection results of tumor tissue‐matched plasma on the TIDE‐Cas14a platform. Weak but statistically significant fluorescence signals were observed in patients P5 and P7 during Cas14a‐ctDNA detection compared to blank controls, whereas ddPCR‐ctDNA missed these two positive samples (P5, P7) (Figure S11b, Supporting Information). This enhanced sensitivity holds significant clinical importance, as early‐stage tumors and minimal residual disease (MRD) typically release ctDNA at concentrations below the detection thresholds of current technologies. For example, as shown in Tables S3 and S4 (Supporting Information), plasma ctDNA from two patients (both at clinical stage IA) was classified as wild‐type by ddPCR but was detectable by TIDE‐Cas14a despite weak fluorescence signals, suggesting its potential utility in identifying early‐stage disease progression.

Based on these results, we speculate that the missed detection by ddPCR‐ctDNA may be related to the following factors: potential fluctuations in ddPCR droplet generation, especially in low‐concentration samples, leading to significant copy number variations between replicates; Degradation of ctDNA samples due to retesting not being performed simultaneously; Poisson distribution of target molecules in droplets, where some droplets may not contain sufficient targets, resulting in signals below the detection threshold; Variations in PCR amplification efficiency due to reaction conditions (such as temperature fluctuations and reagent batch differences), affecting mutation signal detection; TIDE‐Cas14a's reliance on Cas14a's ssDNA recognition capability, which may be more tolerant to fragmented targets; Room for optimization in ddPCR primer‐probe systems. These results demonstrate that Cas14a‐ctDNA detection sensitivity is superior to ddPCR‐ctDNA (100% versus 60%), and confirm the potential of the TIDE‐Cas14a biosensor for ctDNA detection in liquid biopsy applications.

### 2.6 TIDE‐Cas14a‐Based Digital Microfluidic Chip for Ultrasensitive Clinical Molecular Diagnostics

**Figure 7:**
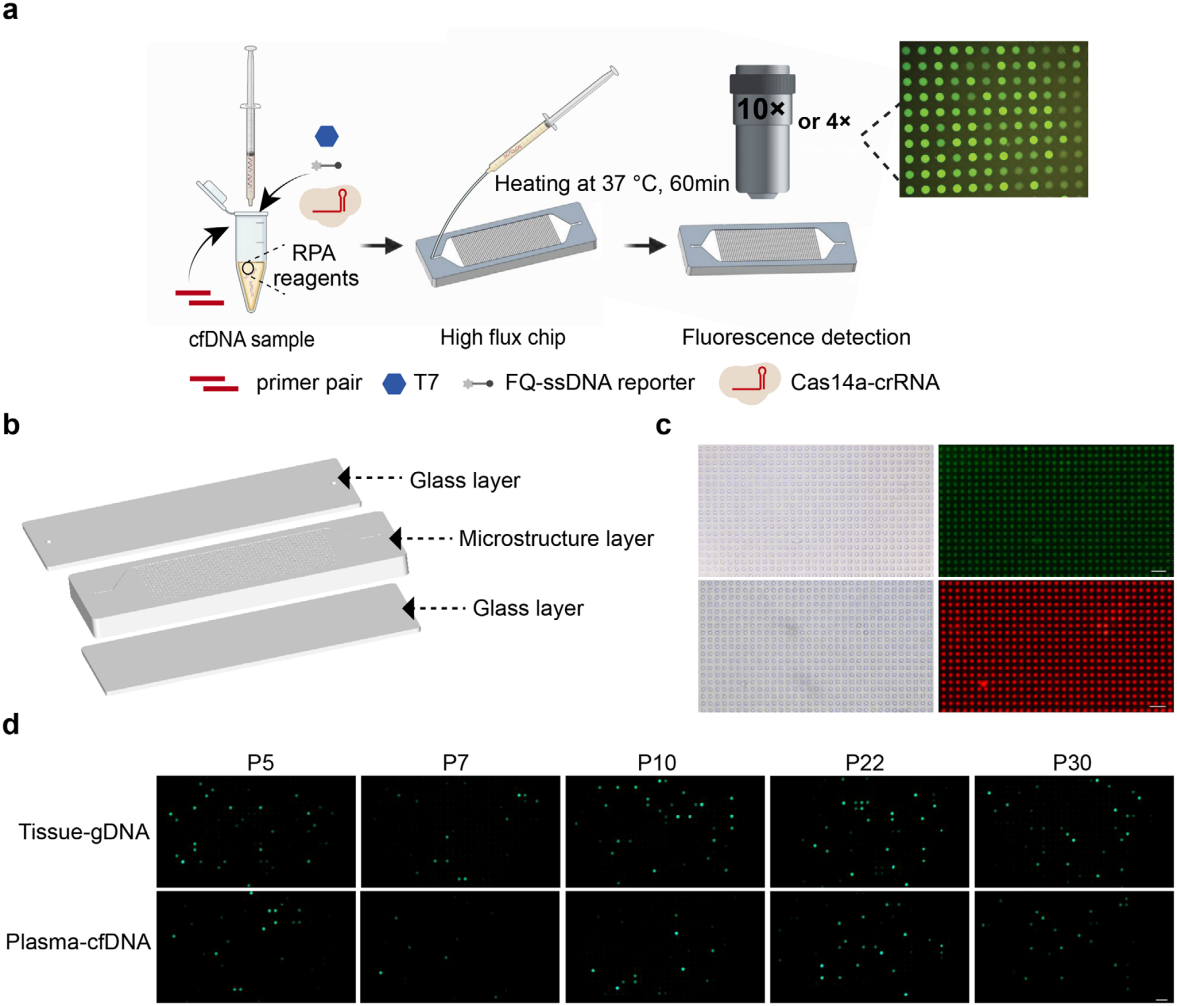
TIDE‐Cas14a system coupled with a digital microfluidic chip. a) Schematic of TIDE‐Cas14a detection system based on digital microfluidic chip. Created in https://BioRender.com b) The 3D architecture of the chip. c) Validation of digital microfluidic chip filling efficiency. Top: Bright‐field and fluorescence images of the chip before and after loading the post‐reaction fluorescent liquid from TIDE‐Cas14a target detection. Bottom: Bright‐field and fluorescence images of the chip before and after ROX dye loading. d) Representative endpoint fluorescence microscopy images of PIK3CA H1047R mutation detection in patient samples using the TIDE‐Cas14a system on a microfluidic chip platform. Scale bars are 100 µm.

Conventional quantitative analysis methods for tumor mutations often suffer from insufficient diagnostic sensitivity and high clinical implementation costs. To address these limitations, we integrated the TIDE‐Cas14a detection method with a digital microfluidic chip independently developed by our laboratory, constructing a microfluidic digital CRISPR detection platform capable of direct and absolute quantification of PIK3CA H1047R mutations in plasma samples (**Figure** **7**a–c; Figure S12, Supporting Information). The digital microfluidic detection chip consists of upper and lower glass layers with an intermediate microstructured body layer. The upper glass sheet is equipped with liquid inlet and waste liquid outlet holes. The chip body layer contains a containment pool comprising an inlet zone, waste liquid zone, and a microcavity array between them. The first glass sheet and the containment pool of the chip body layer form a height‐limited transverse open flow channel, enabling the detection solution added through the inlet hole to rapidly reach the microcavity array via siphon effect under negative pressure, with fast liquid intake and minimal risk of clogging. The chip body layer is fabricated by casting a mixture of PDMS material and curing agent onto a prefabricated mold for replica molding. The resulting microstructure contains an exceptionally high density of microcavities, reaching 100000 microwells, which enables high‐throughput, high‐accuracy, and high‐sensitivity detection of nucleic acid molecules. It should be noted that the current chip has not yet reached the maturity level of commercial digital PCR systems, precluding absolute quantification through Poisson statistics. As an interim solution, we implemented a semiquantitative grading strategy (categorized as “strong positive/weak positive/negative” based on visual assessment of fluorescence intensity clusters) for clinical sample evaluation. To evaluate the feasibility of this method in high wild‐type DNA background conditions, we extracted DNA from 32 tissue and paired plasma samples of BC patients and performed TIDE‐Cas14a reactions in the microfluidic chips (Tables S3 and S4, Supporting Information). As shown in Figure 7c, the chip detection results based on fluorescent microwell counts showed excellent concordance with the one‐pot TIDE‐Cas14a detection results, successfully identifying 5 PIK3CA H1047R mutation‐positive samples. Collectively, these results demonstrate the flexibility and real‐time detection potential of the TIDE‐Cas14a detection system.

## 3 Conclusion

Developing highly sensitive and specific liquid biopsy platforms to precisely capture low‐abundance somatic mutations in plasma ctDNA (such as driver gene alterations in EGFR, ALK, etc.), while integrating innovative technologies like single‐molecule sequencing, microfluidic enrichment, or AI‐assisted analysis, has emerged as a critical frontier in advancing precision oncology.^[^ ^24^, ^64^, ^65^, ^66^, ^67^, ^68^ ^]^ In this study, we developed TIDE‐Cas14a, creating an optimized RPA‐T7‐CRISPR/Cas14a system for the ultrasensitive detection of the critical BC driver mutation PIK3CA H1047R within 1 h at 37 °C.^[^ ^26^, ^52^ ^]^ This platform integrates the rapid amplification capability of RPA, the strand‐specific digestion property of T7 exonuclease, and the ssDNA cleavage specificity of CRISPR/Cas14a. By introducing synthetic G/C base mismatches at specific positions in the crRNA, we enhanced the system's SNP discrimination capability, achieving a detection limit as low as 0.01% VAF that was significantly more sensitive than ddPCR (LOD: 0.1%).^[^ ^69^, ^70^ ^]^ Compared to ddPCR, our platform demonstrates notable advantages in simplicity, speed, accuracy, and low cost. When benchmarked against CRISPR‐based SNP detection methods reported in the CASMART study (Table S2, Supporting Information), TIDE‐Cas14a represents one of the few platforms capable of achieving high‐sensitivity SNP detection.^[^ ^23^ ^]^

We further adapted the TIDE‐Cas14a system to our laboratory's proprietary 100000‐well microfluidic chip. Given the low fluidity characteristics of RPA reaction reagents due to their multi‐enzyme system, our microfluidic chip's key advantage lies in its well size that permits RPA detection reagents to enter the microwells while maintaining sufficient well density to ensure both sensitivity and resolution for ctDNA detection. This design enables near single‐molecule sensitivity in digital detection mode with reduced sample requirements. The platform demonstrates a remarkable 0.001% VAF detection capability, surpassing optimized conventional reaction systems while retaining the benefits of isothermal amplification and rapid turnaround. This dual‐mode compatibility (bulk reactions for initial screening and digital analysis for confirmation) establishes TIDE‐Cas14a as a versatile tool for clinical staging applications requiring both high sensitivity and quantitative precision. The high concordance between plasma ctDNA analysis and matched tissue biopsy results validates its potential for noninvasive monitoring of tumor evolution. These findings position TIDE‐Cas14a as a transformative technology that provides a new paradigm for noninvasive cancer monitoring and early diagnosis, particularly suited for POCT applications. Notably, TIDE‐Cas14a outperforms previously reported CRISPR‐based diagnostic methods in terms of SNP detection sensitivity (Table S2, Supporting Information). Moreover, the chip‐based TIDE‐Cas14a platform offers advantages such as short turnaround time, ease of operation, and low cost, making it a promising candidate for future quantitative SNP analysis (Table S5, Supporting Information). However, it should be noted that the current design relies on a visual “strong/weak positive” classification, which limits its diagnostic precision in applications such as minimal residual disease monitoring or treatment response assessment. To further enhance the quantitative capability of the system, based on previous studies such as the CASMART method,^[^ ^23^, ^71^, ^72^, ^73^ ^]^ we believe that Poisson statistics could indeed be applicable to our system. Nonetheless, certain technical, resource and time‐related constraints currently pose challenges to fully realizing this capability. Therefore, we plan to continue exploring this direction in future studies and gradually optimize the current design.

While TIDE‐Cas14a represents a significant advancement, several challenges warrant further investigation. First, the current validation was conducted in a single‐center cohort with limited sample size (n = 32). Multicenter studies involving larger prospective cohorts are required to establish clinical validity and define optimal VAF thresholds for therapeutic decision‐making. Second, the platform's performance in detecting mutations in non‐plasma samples (e.g., cerebrospinal fluid, urine) has not been explored, its applicability across different cancer types is insufficiently demonstrated, and systematic comparison with established methods remains incomplete. The practical utility of this technology in real‐world clinical settings still requires further validation. Given the growing interest in alternative liquid biopsy matrices, expanding the assay's compatibility could further broaden its clinical applications. Furthermore, relying exclusively on ddPCR as the benchmarking method entails certain limitations.^[^ ^15^, ^16^ ^]^ First, although ddPCR excels in quantitative mutation detection, it is predominantly effective for targeted screening of known mutation sites. Second, ddPCR may encounter difficulties when analyzing complex samples, such as detecting low‐frequency mutations in the presence of a high wild‐type background. Variability during the droplet generation process can compromise reproducibility, particularly in low‐concentration samples. And a critical limitation of current ctDNA analysis lies in the prerequisite DNA extraction/enrichment steps required to overcome low VAF constraints. It is noteworthy that while TIDE‐Cas14a demonstrates excellent performance in detecting PIK3CA H1047R mutations, its potential for multiplex nucleic acid detection remains unexplored. Additionally, optimization of crRNA mismatch positions and base types (A/T) requires mutation‐specific customization, representing another critical area for improvement. Future iterations could fully leverage Cas14a's multiplex crRNA targeting capability to enable simultaneous detection of multiple mutations in a single reaction, thereby increasing diagnostic yield and better capturing tumor heterogeneity. Of particular significance, our future work will focus on integrating the TIDE‐Cas14a detection method with our laboratory‐developed digital microfluidic chip and accompanying isothermal detection cartridge. This integrated system, when combined with smartphone‐based fluorescence imaging, could potentially eliminate the need for complex laboratory instruments and achieve true POCT and at‐home testing. Such technological integration would not only enable a “sample‐to‐answer” automated workflow but also, through its portable design, make the platform particularly suitable for primary healthcare settings, providing an innovative solution for early cancer screening and diagnosis.

In conclusion, TIDE‐Cas14a provides a novel platform for the ultrasensitive detection of cancer‐associated mutations in CRISPR‐based liquid biopsies. By integrating isothermal amplification, enzymatic strand selection, and CRISPR activation through optimized crRNA engineering, this platform overcomes the long‐standing sensitivity‐specificity trade‐off that has constrained conventional methodologies. Its demonstrated superiority over ddPCR, combined with rapid operation requiring no sophisticated instrumentation, establishes this system as a transformative solution for early cancer detection, MRD monitoring, and real‐time assessment of therapeutic response. As the field advances toward increasingly personalized treatment paradigms, we contend that TIDE‐Cas14a could make significant contributions to advancing CRISPR/Cas‐assisted diagnostic assays, thereby unlocking the full potential of precision oncology.

## 4 Experimental Section

### Ethics and Sample Inclusion Statement

This study was approved by the Research Ethics Committee of Harbin Medical University Cancer Hospital (KY 2022‐65) and conducted following the principles of the Declaration of Helsinki. Patients were recruited from Harbin Medical University Cancer Hospital, and individuals with histologically confirmed BC who had not received radiotherapy or chemotherapy before surgical resection were considered eligible for participation in this study. Before study initiation, healthy volunteers were recruited. Individuals with no history of cancer and negative screening results were deemed healthy. All patients and healthy volunteers provided written informed consent before specimen donation.

### Patient Samples

A total of 32 BC patient tissue and paired plasma samples, along with 30 healthy volunteer plasma samples, were enrolled in this study. gDNA was extracted from BC tissues using the genomic DNA extraction kit (Tiangen Biotech Co., Ltd., Beijing, China). Briefly, tissues were homogenized into cell suspensions, followed by centrifugation at 10000 rpm (≈11200×g) for 1 min. Subsequently, 200 µL of Buffer GA was added and vortexed until complete suspension. Then, 4 µL of RNase A (100 mg mL^−1^) (Cat No. RT405‐12, Tiangen Biotech Co., Ltd., Beijing, China) was added, vortexed for 15 s, and incubated at room temperature for 5 min to eliminate RNA contamination. Next, 20 µL of Proteinase K (20 mg mL^−1^) (Cat No. RT403‐02, Tiangen Biotech Co., Ltd., Beijing, China) was added and incubated at 56 °C for 2 h. After adding 200 µL of Buffer GB, samples were incubated at 70 °C for 10 min. Sequential washing was performed with 200 µL of absolute ethanol, 500 µL of Buffer GD, and 600 µL of Buffer PW, with centrifugation at 12000 rpm (≈13400×g) for 30 s each. Following a 2‐min air‐drying at room temperature, gDNA was eluted with 100 µL of TE elution buffer to obtain purified gDNA solutions. DNA concentration was determined by absorbance at 260 nm using a NanoDrop One spectrophotometer (Thermo Fisher Scientific, Waltham, MA, USA), and samples were stored at −20 °C.

Whole blood was collected in cell‐free DNA preservation tubes (Ardent BioMed, Guangzhou, China) and processed within one day of storage at 4 °C. Initially, samples were centrifuged at 1600 ×g for 10 min at 4 °C to pellet cellular components. The upper plasma layer was carefully transferred to new 2 mL microcentrifuge tubes. Subsequently, plasma was centrifuged at 16000 ×g for 10 min at 4 °C to remove residual cellular debris. The supernatant was stored at −80 °C until DNA extraction. To meet the requirements of ddPCR and RPA‐T7‐CRISPR/Cas14a assays, cfDNA was extracted from a minimum of 4 mL plasma per sample whenever possible. cfDNA was isolated from 4 mL plasma using the QIAamp Circulating Nucleic Acid Kit (Cat No. 55 114, Qiagen GmbH, Hilden, Germany) following sequential steps of lysis, binding, washing, and elution. Each 4 mL plasma sample was eluted with 70 µL of AVE Buffer and stored at −80 °C. The 70 µL cfDNA solution was lyophilized using a freeze‐dryer to obtain cfDNA powder for concentration enhancement. cfDNA powder could be stored at −80 °C. For ddPCR or RPA‐T7‐CRISPR/Cas14a reactions, the lyophilized product was reconstituted in 10 µL of ddH2O.

### Primer and DNA Fragments Preparation

All HPLC‐purified oligonucleotide sequences and ssDNA reporter probes labeled with different fluorophores were synthesized by Sangon Biotech Co., Ltd (Shanghai, China), with sequences detailed in Table S1 (Supporting Information). Both mutant (PIK3CA H1047R) and wild‐type dsDNA fragments were synthesized and cloned into pUC57 vectors by Hongxun Biotechnologies Co., Ltd (Suzhou, China). For PCR amplification, a 50 µL reaction system was prepared containing plasmid as the template, 25 µL Premix Taq (Cat No. R401A, Takara Bio Inc., Dalian, China), and 480 µM forward/reverse primers. Amplification was performed on a ProFlex 3 x 32‐well PCR System (Cat No. 4484073, Thermo Fisher Scientific, Waltham, MA, USA) using the following thermal cycling parameters: 30 cycles of 98 °C for 10 s (denaturation), 55 °C for 30 s (annealing), and 72 °C for 1 min (extension). PCR products were quantified using a NanoDrop One spectrophotometer and stored at ‐80 °C.

### Design of RPA Primers

The RPA primers were designed targeting the conserved region of the PIK3CA H1047R gene using Primer Premier 6 software (Premier Biosoft International, Palo Alto, CA, USA), with specificity validation performed through MFEprimer 4.0 (https://m4.igenetech.com/),^[^ ^74^ ^]^ while OligoAnalyzer (IDT, Coralville, IA, USA; https://www.idtdna.com) was employed for Tm calculation and secondary structure prediction. The forward RPA primer was modified with PT modification at the first four 5′ nucleotides to prevent T7 exonuclease degradation in subsequent detection steps, whereas the reverse primer remained unmodified. Primer specificity and RPA amplification product generation were verified by 3% agarose gel electrophoresis (Cat No. A4718, Sigma‐Aldrich, St. Louis, MO, USA), with DNA Marker (Cat No. SM0373, Thermo Fisher Scientific, Waltham, MA, USA) and various amplicons electrophoresed in 1×TBE running buffer (Cat No. BL548A, Biosharp, Hefei, China) at 5 V cm^−1^ for 1 h. Following electrophoresis, the gel was completely immersed in a staining solution prepared by mixing 100 mL of 1×TBE running buffer with 10 µL SYBR Green I (Cat No. K1004, MedChemExpress, China), followed by gentle agitation at room temperature in the dark for 30–60 min. After staining, gel visualization was performed using a gel imaging system (ProteinSimple, San Jose, CA, USA).

### In Vitro Transcription and Purification of sgRNA

The CRISPR/Cas14a‐sgRNA sequences were designed based on previously reported studies.^[^ ^52^ ^]^ Initially, we prepared the sgRNA transcription template using manually designed primers including SgRNA‐F and SgRNA‐R targeting the PIK3CA H1047R mutant sequence, where a 200 µL microcentrifuge tube reaction mixture containing 10 µL PCR Premix Taq DNA polymerase (Cat No. R004A, Takara Bio Inc., Dalian, China), 0.5 µL SgRNA‐F (10 µM), 0.5 µL SgRNA‐R (10 µM), and 9 µL DEPC‐treated water was subjected to PCR amplification and annealing under the following conditions: initial denaturation at 95 °C for 2 min; 35 cycles of 95 °C for 15 s, 50 °C for 15 s, and 72 °C for 10 s; final hold at 25 °C, yielding the CRISPR/Cas14a‐sgRNA DNA template containing the T7 promoter (sequences provided in Table S1, Supporting Information). Subsequently, in vitro transcription of CRISPR/Cas14a‐sgRNA was performed using the High Yield crRNA Synthesis and Purification Kit (Cat No. 31903, Tolo Biotech Co., Ltd, Shanghai, China), where a 20 µL reaction system containing 4 µL 5× TranscriptMax Reaction Buffer, 8 µL NTP mix, 2.1 µL TranscriptMax Enzyme Mix, 5 µL PCR products, and 0.9 µL DEPC‐treated water was incubated at 37 °C for 12‐16 h, followed by addition of 25 µL 2× DNase I Buffer and 4 µL DNase I with 1 µL DEPC‐treated water and incubation at 37 °C for 30 min to eliminate residual DNA templates, with final purification of transcription products performed according to the manufacturer's protocol and resuspension in DEPC‐treated water.

### RPA‐T7‐CRISPR/Cas14a Detection

The RPA‐T7‐CRISPR/Cas14a cleavage reaction was performed using commercially obtained reagents including Cas14a Nuclease, 10× HOLMES Buffer, and HOLMES ssDNA reporter (5′FAM/3′Quencher, FQ‐ssDNA reporter) (Cat No. 31101, Tolo Biotech Co., Ltd, Shanghai, China). For the 20 µL reaction system, the following components were combined: 2 µL 10× HOLMES Buffer, 1.25 µL Cas14a Nuclease (10 µm), 1.25 µL PIK3CA‐mut‐sgRNA (10 µM), 1.25 µL FQ‐reporter (10 µM), 3 µL 5× T7 Gene 6 Exonuclease Buffer, 1 µL T7 Gene 6 Exonuclease (50 U µL^−1^, Cat No. 70025, Thermo Fisher Scientific, Waltham, MA, USA), 10 µL RPA premix, 0.2 µL DTT (10 mm), and 0.05 µL DNase/RNase‐free ddH2O. The reaction mixture was incubated at 37 °C in an Applied Biosystems StepOnePlus Real‐Time PCR System (Thermo Fisher Scientific, Waltham, MA, USA) with fluorescence intensity measurements acquired every minute for 60 cycles (≈1 h total duration). Simultaneous visual detection was performed using a blue light transilluminator (Zeesan Biotech Co., Ltd, Xiamen, China) with 485 nm excitation.

### Chip Imaging and Analysis

Quantification in the digital CRISPR/Cas14a assay was performed using a lab‐developed digital microfluidic chip (Chinese Patent Application Publication No. CN119303644A, 2025). The proprietary microfluidic chip measures 74 mm (length) × 25 mm (width) × 10 mm (height), comprising an upper glass layer, PDMS chip microstructured body layer, and lower glass layer bonded via plasma treatment. Before loading, the chip underwent hydrophilicity/hydrophobicity modification and vacuum treatment. The chip contains 100000 microwells (50 µm diameter, 80 µm depth, 40 µm spacing) with individual well volumes of 0.157 nL, enabling high‐throughput, high‐accuracy, and high‐sensitivity nucleic acid detection.

### The Workflow Proceeded as Follows

1) Chip preparation via 20‐min vacuum treatment using a vacuum pump; 2) Preparation of RPA‐T7‐CRISPR/Cas14a reaction mix in a 200 µL PCR tube containing: 480 nM forward primer, 480 nM reverse primer, rehydration buffer with lyophilized enzyme pellet, 1× HOLMES Buffer, 625 nM Cas14a nuclease, 625 nM PIK3CA‐mut‐sgRNA, 625 nM FQ‐ssDNA reporter (10 µM), 3 µL 5× T7 Gene 6 Exonuclease Buffer, 2.5 U/µL T7 Gene 6 Exonuclease, 10 mM DTT, target DNA at desired concentration, and DNase/RNase‐free ddH2O to 100 µL final volume; 3) Immediate transfer of reaction mix onto the chip after adding 280 nM MgOAc, completing loading within 5 min; 4) Sealing of microwells with FC‐40 fluorinated oil and removal of excess liquid.^[^ ^62^ ^]^ All procedures were performed on ice (Figure S12, Supporting Information).

Loaded chips were incubated at 37 °C for 60 min in a ProFlex PCR System. Endpoint fluorescence signals were visualized using an SOPTOP ICX‐41 inverted fluorescence microscope (Sunny Optical Technology Co., Ltd, China) with 350 ms exposure time. Captured images were analyzed using ImageJ software (National Institutes of Health, Bethesda, MD, USA) for automated positive droplet quantification.

### Statistical Analysis

Statistical processing and visualization were performed using GraphPad Prism 9.0 (GraphPad Software, San Diego, CA, USA), and ImageJ software. Unless otherwise specified, all experiments were conducted with three technical replicates, and results are expressed as mean ± standard deviation (s.d.). Between‐group comparisons were performed using two‐tailed Student's t‐tests, and statistical significance was defined as *p* < 0.05. For multigroup comparisons, one‐way analysis of variance (ANOVA) was applied. Standard curves were generated through nonlinear regression.

## Conflict of Interest

The authors declare no conflict of interest.

## Author Contributions

Y.Y. and M.J. contributed equally to this work. Y.Y., X.Z., Y.G., and W.Y. conceived and designed the project. Y.Y. developed and optimized the detection system. W.Y., Y.G., and S.L. performed the theoretical calculations. X.Q., J.H., W.H., and Q.F. collected clinical samples. M.J., J.L., S.L., and F.F. were responsible for the handling of clinical samples. Y.Y., M.J., H.L., Y.C., and Y.W. conducted the laboratory‐related experimental research. Y.Y. edited the manuscript. X.Z., D.P., J.L., and Y.Z. provided the detection concept, writing guidance, and project supervision. X.Z. provided the financial support. All authors provided feedback and reviewed the manuscript.

## Supporting information



Supporting Information

Supporting Information

## Data Availability

The data that support the findings of this study are available in the supplementary material of this article.
