# Supplementary material for: Engineered crRNA Drives RPA‐T7‐CRISPR/Cas14a Cascade for Ultrasensitive Detection of ctDNA PIK3CA H1047R
Source: Adv Sci (Weinh). 2025 Aug 30;12(44):e07126. doi: 10.1002/advs.202507126 (PMC12667487; doi:10.1002/advs.202507126)
Supplement: Supplementary file 1 — Supporting Information [file ADVS-12-e07126-s002.docx]

Supporting information

**Engineered crRNA Drives RPA-T7-CRISPR/Cas14a Cascade for Ultrasensitive Detection of ctDNA PIK3CA H1047R**

**Authors**

Yuanyuan Yu^1^, Mengru Jin^1^, Weiguang Yuan^2^, Yajie Gong^1^, Siwei Li^1^, Xuquan Qin^1^, Jianxun Hou^1^, Jialin Liu^1^, Siyu Liu^1^, Hui Li^1^, Yijun Chu^1^, Yingjie Wang^1^, Youxue Zhang^1^, Fang Fang^1^, Wenhui Hao^1^, Yuling Gu^3^, Qinchen Fan^1^, Jing Lin^2^*, Da Pang^1^*, Xianyu Zhang^1^*

^1^Department of Breast Surgery, Harbin Medical University Cancer Hospital, Harbin, Heilongjiang Province, 150086, China.

^2^Institute of Cancer Prevention and Treatment, Harbin Medical University, Heilongjiang Academy of Medical Sciences, Harbin, Heilongjiang Province, 150086, China.

^3^Shanghai Naturethink Life & Scientific Co., Ltd.

Author Contributions: Yuanyuan Yu and Mengru Jin contributed equally to this work.

*Corresponding author E-mail: [alin-00@163.com](mailto:alin-00@163.com); [pangda@ems.hrbmu.edu.cn](mailto:pangda@ems.hrbmu.edu.cn); [zhangxianyu@ems.hrbmu.edu.cn](mailto:zhangxianyu@ems.hrbmu.edu.cn)

**1. Experimental Methods**

**gDNA extraction:** Cell lines were selected based on PIK3CA mutational subtypes, including the human breast cancer T-47D cell line (Procell, China) (positive for PIK3CA H1047R mutation), MCF-7 cell line (Procell, China) (positive for PIK3CA E545K mutation), CAL-51 cell line (MeisenCTCC, Zhejiang, China) (positive for PIK3CA E542K mutation), H1975 cell line (Procell, China) (positive for EGFR T790M mutation), H3255 cell line (MeisenCTCC, Zhejiang, China) (positive for EGFR L858R mutation), HT-29 cell line (Procell, China) (positive for BRAF V600E mutation), SW480 cell line (Procell, China) (positive for KRAS G12V mutation) and MDA-MB-231 cell line (Procell, China) (wild-type control for PIK3CA H1047R), from which gDNA was extracted. The Variant Allele Frequency (VAF) of PIK3CA in these cell lines have been confirmed by the Catalogue of Somatic Mutations in Cancer (COSMIC) database, Cell Model Passports database, and previous experiments.^[1]^ The T-47D , H1975 and H3255 cell lines was cultured in RPMI-1640 medium (Gibco, USA) supplemented with 10% fetal bovine serum (FBS; Cat No. ST30-3302P, PAN-Biotech GmbH, Germany), 1% penicillin-streptomycin (P/S; Gibco, USA), and 10 μg/mL insulin, while the MCF-7 cell line was maintained in MEM medium (Gibco, USA) containing 10% FBS, 1% P/S, and 10 μg/mL insulin (Cat No. P3378, Beyotime Biotechnology Co., Ltd, Shanghai, China). CAL-51, SW480 and MDA-MB-231 cell lines were cultured in DMEM medium (Gibco, USA) supplemented with 10% FBS and 1% P/S. HT-29 cell line was cultured in McCoy's 5A medium (Gibco, USA) containing 10% FBS and 1% P/S. All cell lines were cultured at 37°C in either a humidified incubator with 5% CO₂ and 95% air or an ambient air incubator. gDNA was extracted from cell lines using the genomic DNA extraction kit following a similar procedure to that used for tissue gDNA extraction, strictly according to the manufacturer’s instructions, with final elution performed using 100 μL of TE buffer. The gDNA concentration was determined by measuring absorbance at 260 nm using a NanoDrop One spectrophotometer, adjusted to an initial concentration of 100 ng/μL, and stored at -80°C until use.

**RPA reaction:** The RPA reaction system was established according to the TwistAmp Basic Kit Quick Guide protocol, where 29.5 μL rehydration buffer, 4.8 μL forward primer (10 μM), 4.8 μL reverse primer (10 μM), and 7.4 μL DNase/RNase-free ddH2O were initially added to the PCR tube containing the pre-mixed enzyme pellet and thoroughly mixed to form the RPA master mix. Subsequently, 1 μL of dsDNA template (100 ng/μL) and 2.5 μL MgOAc (280 mM, provided in kit) were added to the reaction system, followed by vortex mixing and brief centrifugation to initiate the reaction, with incubation performed at 39°C for 20 minutes using a ProFlex™ 3 × 32-well PCR System. To determine optimal RPA conditions, 50 μL reaction systems were tested across various parameters, including incubation durations (10, 20, 30, and 40 minutes) and temperatures (35°C, 37°C, 39°C, and 41°C), with all experimental conditions maintained in technical triplicates.

**ddPCR assay:** For ddPCR experiments, sequence-specific primers and probes targeting the PIK3CA H1047R gene were designed (sequences provided in Supplementary Materials Table S1), following the same design methodology as the RPA primers. The mutant probe was 5’-labeled with 6-carboxy-fluorescein (FAM), while the wild-type probe was 5’-labeled with hexachloro-fluorescein (HEX). Before the patient sample analysis, the analytical performance of the ddPCR assay was evaluated using breast cancer cell line gDNA (T-47D and MDA-MB-231). The ddPCR detection kit (Turtle Tech Co., Ltd, Shanghai, China) was employed to prepare 30 μL reaction mixtures containing: 10 μL 3× Maxuseful PCR Buffer, 0.5 μL Taq DNA polymerase (5 U/μL), 1.2 μL forward primer (10 μM), 1.2 μL reverse primer (10 μM), 0.6 μL FAM-labeled probe (10 μM), 0.6 μL HEX-labeled probe (10 μM), 1 μL target gDNA (100 ng/μL), and 15.9 μL DNase/RNase-free ddH2O. All reactions were performed on the BioDigital-Qing DPCR™ System (Turtle Tech Co., Ltd, Shanghai, China) using the following workflow. First, automated droplet generation was performed using the BioDigital-QING Loader™. Specifically, 2 mL of Oil A and 1 mL of Oil B were loaded into reservoirs A1 and B1, respectively. This oil system supports 24 simultaneous reactions. Second, amplification was performed on the BioDigital-QING Cycler™ using the following protocol: 50°C for 5 min, 95°C for 5 min, 50 cycles of 95°C for 15 s and 63°C for 30 s, followed by a final hold at 25°C. Furthermore, fluorescent signals were detected and data were acquired using the BioDigital-QING Imager™. Negative controls consisted of ddH2O blanks subjected to the same processing as test samples. To optimize annealing temperature, we tested parallel 30 μL reactions at 62°C, 63°C, and 64°C during amplification.

**Linear range test and judgment of LOB and LOD:** To comprehensively evaluate the performance of the established RPA-T7-CRISPR/Cas14a system, this study assessed three critical parameters including linear range, LOB, and LOD, with sensitivity comparisons made against the ddPCR platform, where the LOB was determined by analyzing false-positive events from 30 consecutive measurements of healthy donor peripheral blood cfDNA samples with measurements below the LOB threshold classified as negative indicating no statistically significant target signal. For linear range and LOD assessment, serial dilutions of PIK3CA H1047R mutant cell line (T-47D) gDNA (initial concentration: 100 ng/μL) were prepared using DNase/RNase-free ddH2O to generate concentrations of 100, 50, 40, 30, 20, 10, 1, 0.1, and 0.01 ng/μL. Separately, mixed DNA templates with VAFs of 50%, 10%, 1%, 0.1%, 0.01%, and 0.001% were prepared by blending PIK3CA H1047R mutant and wild-type gDNA (both at 100 ng/μL; MDA-MB-231 cell line gDNA served as WT control, see Supporting Information for details), using the T-47D cell line (heterozygous mutation, VAF≈50%) as reference,^[1]^ with specific 100 μL total volume mixtures prepared as follows: 50% VAF (100 μL MT + 0 μL WT), 10% VAF (20 μL MT + 80 μL WT), 1% VAF (2 μL MT + 98 μL WT), 0.1% VAF (0.2 μL MT + 99.8 μL WT), 0.01% VAF (0.02 μL MT + 99.98 μL WT), and 0.001% VAF (0.002 μL MT + 99.998 μL WT). All dilution series and VAF standards were tested in triplicate (n=3) using the RPA-T7-CRISPR/Cas14a system with either synthetic fragments or cell line gDNA from different batches, where fluorescence signals (ΔF) were recorded every 30 seconds to generate dose-response curves, and linear ranges were determined by analyzing the correlation between ΔF and log10(VAF), while for ddPCR comparisons, quadruplicate samples (n=4) were analyzed with linear ranges determined by correlating log10(theoretical copy numbers) versus log10(measured copy numbers), and the LOD was defined as the lowest concentration or VAF where the signal value or copy number exceeded the LOB at 95% confidence level.

**Molecular docking study of the Cas14a/sgRNA-Target ssDNA complex:** The HDOCK webserver (http://hdock.phys.hust.edu.cn/) was used for docking calculations.^[2,3]^ Potential binding modes were searched using a fast Fourier transform algorithm and evaluated using a knowledge-based scoring function. The top 10 docking results were visualized, and the top 100 results were provided for download. The docking combination with the most negative score was selected for interaction analysis using PyMOL. Docking scores were calculated using the knowledge-based iterative scoring function ITScorePP or ITScorePR; more negative scores indicate more likely binding models.

**2. Supplementary Figures**


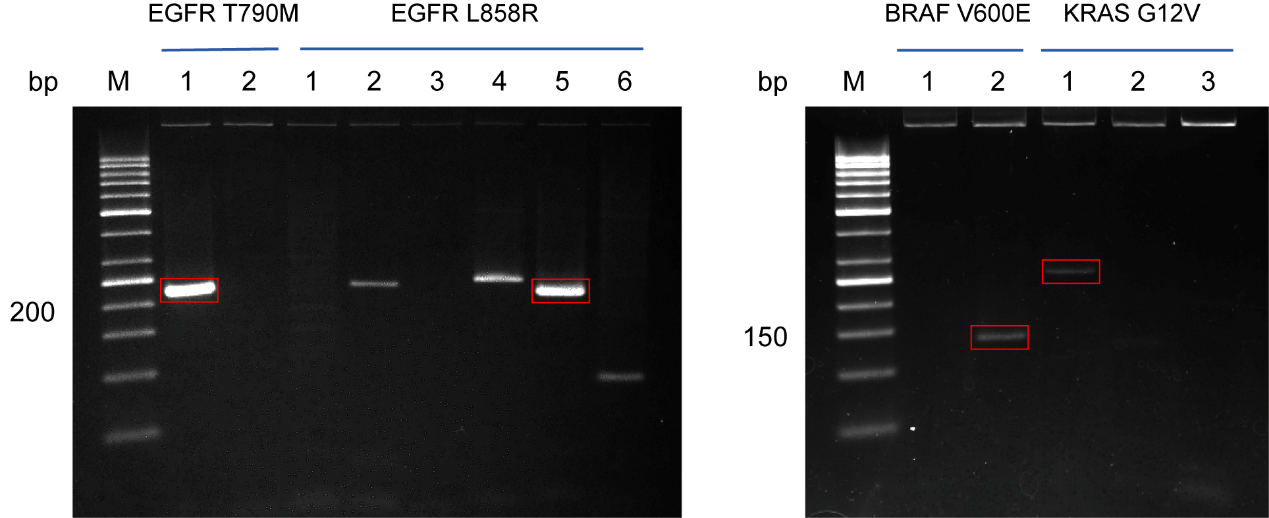


**Figure S1.** Agarose gel electrophoresis result for optimization of RPA amplification primers targeting EGFR T790M, EGFR L858R, BRAF V600E and KRAS G12V mutations. Source data are provided as a Source Data file.


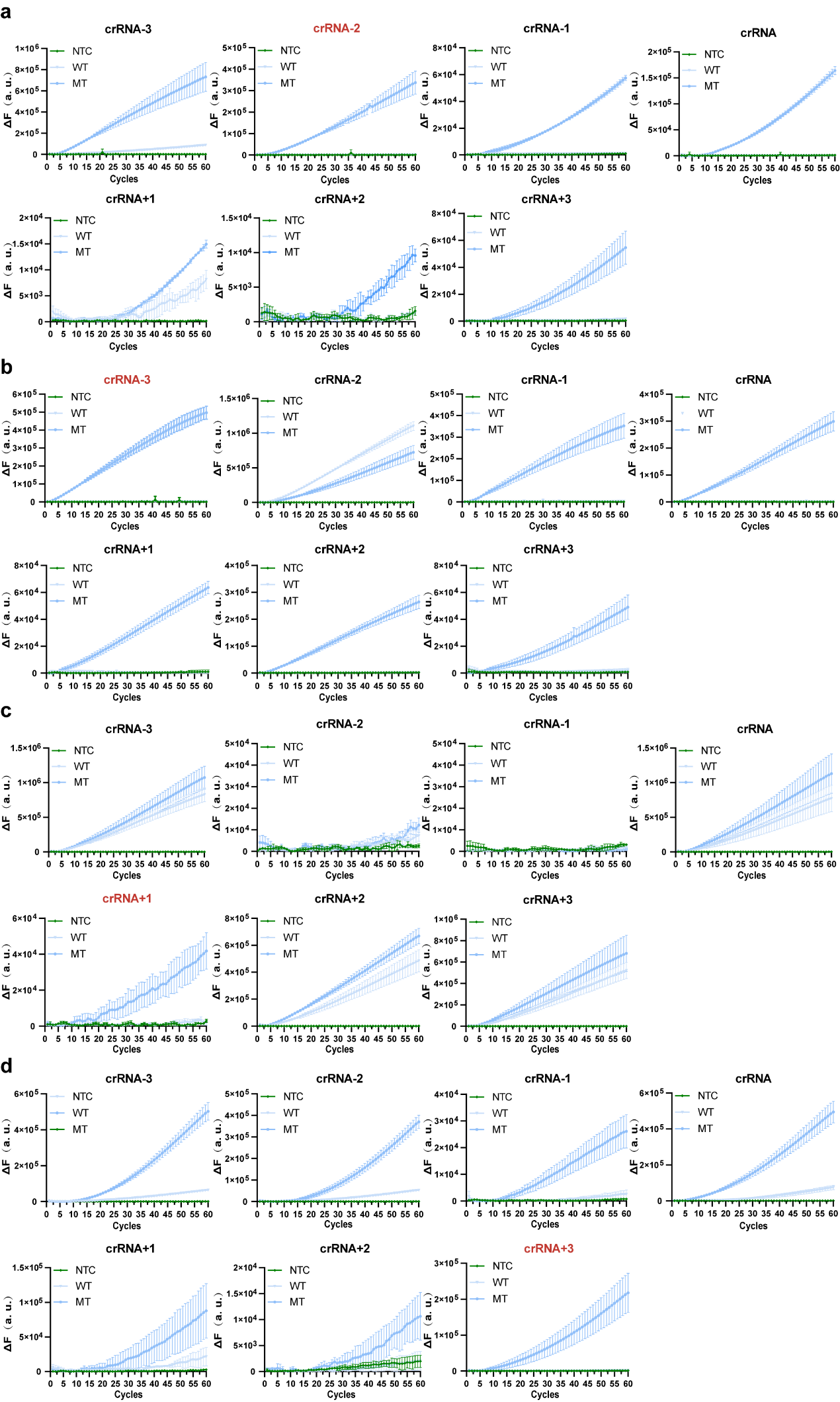


**Figure S2.** Real-time fluorescence curves​​ demonstrating the ​​specificity of crRNA screening​​ for ​​EGFR T790M​​, ​​EGFR L858R​​, ​​​​BRAF V600E​​​​, and KRAS G12V mutations detection. NTC, no-template control. WT, wild template. MT, mutant template. Data represent the means ± standard deviation (s.d.) (n = 3). Source data are provided as a Source Data file.


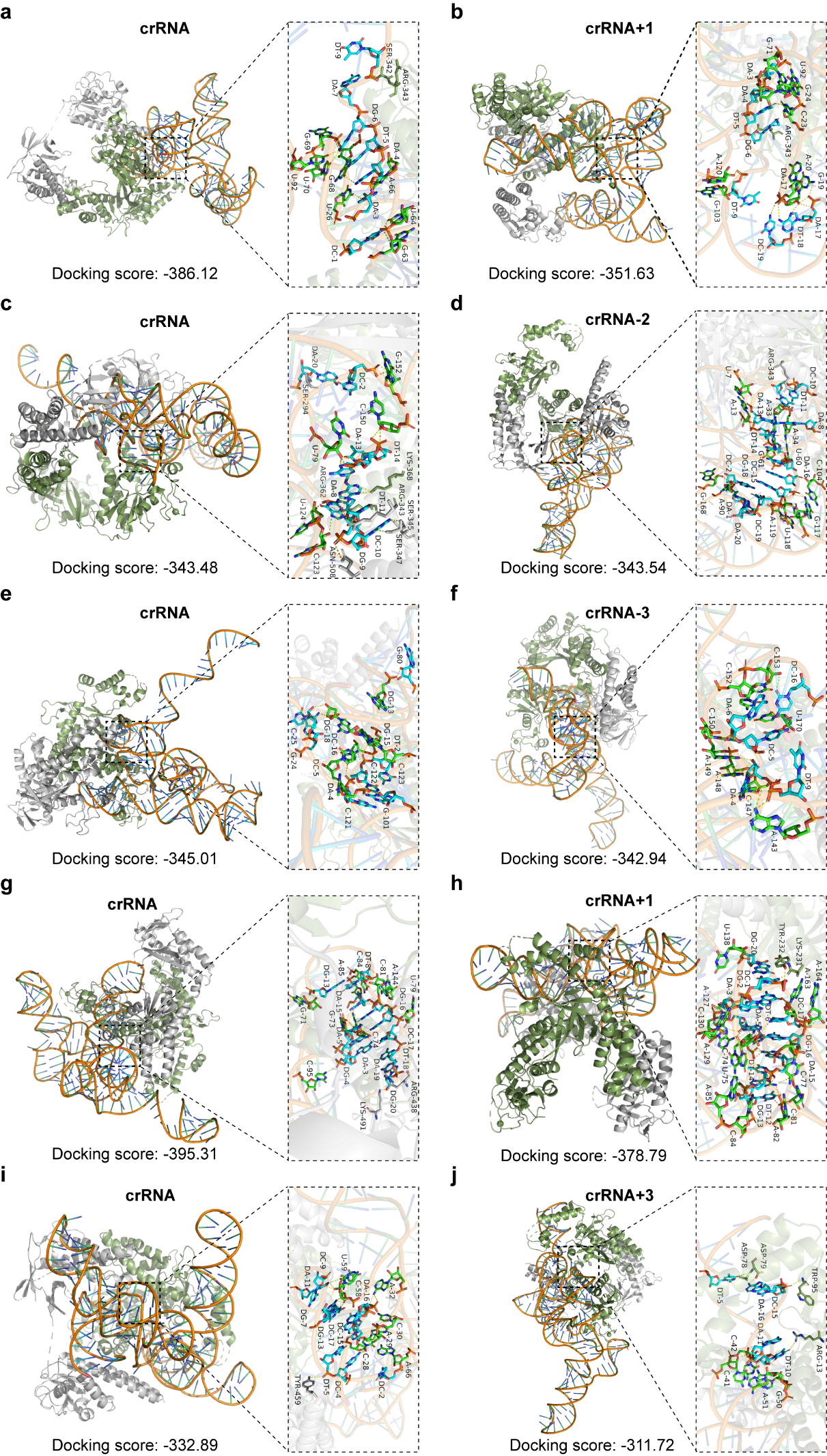


**Figure S3.** Molecular docking calculations of Cas14a interacting with sgRNA and target DNA. Docking results of wild-type PIK3CA H1047R, EGFR T790M, EGFR L858R, BRAF V600E, and KRAS G12V genes with Cas14a in complex with the original crRNA and the the selected best specificity crRNA.


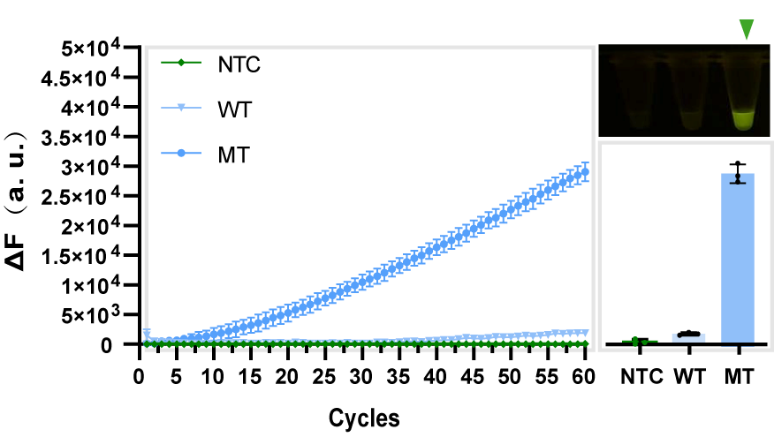


**Figure S4.** Feasibility validation of an RPA-T7-CRISPR/Cas14a biosensor for detecting the PIK3CA H1047R mutation in breast cancer. NTC, no-template control. WT, wild template. MT, mutant template. Data represent the means ± standard deviation (s.d.) (n = 3). Source data are provided as a Source Data file.

**
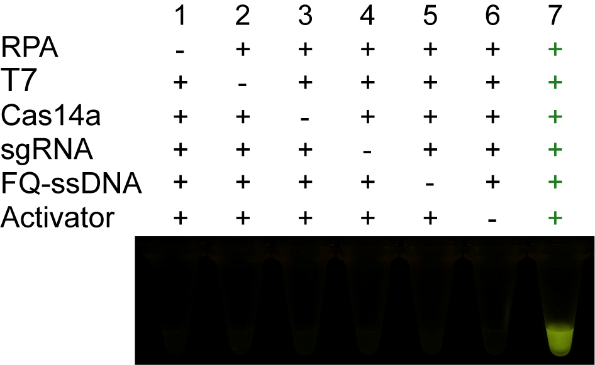
**

**Figure S5.** Validation of the TIDE-Cas14a system. The six reaction groups individually lacked RPA amplification substrate, T7 exonuclease, Cas14a, sgRNA, FQ-ssDNA reporter, and target DNA. Only Reaction 7, containing all six required components, generated a fluorescent signal. Data represent the means ± standard deviation (s.d.) (n = 3). Source data are provided as a Source Data file.

**
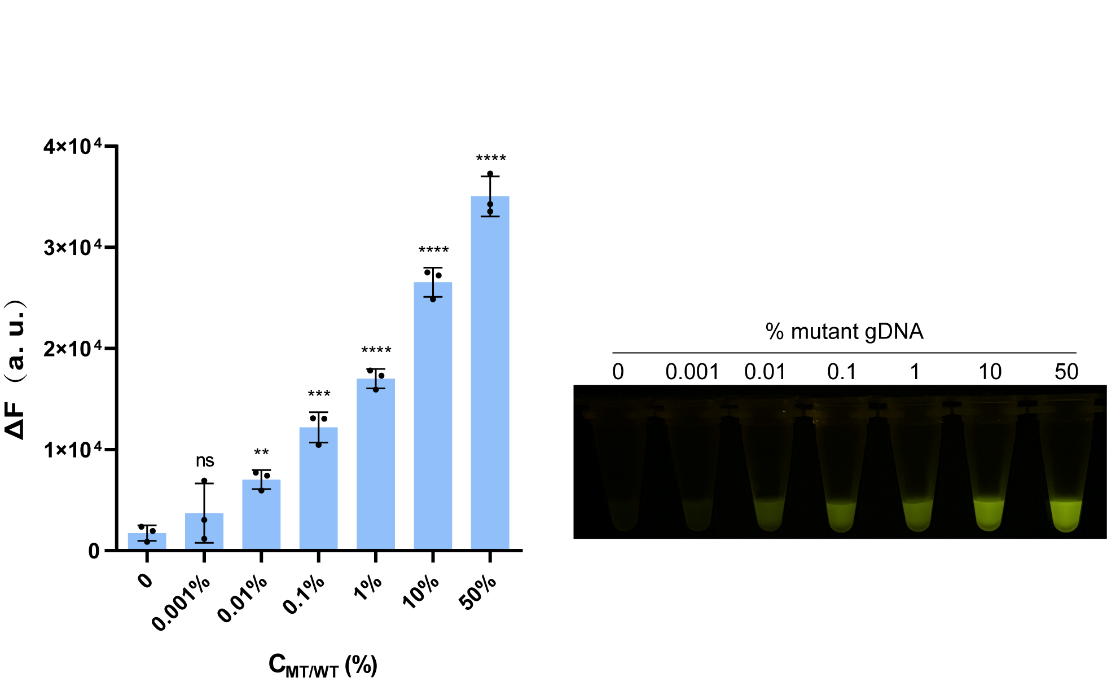
**

**Figure S6.** Endpoint fluorescence values of PIK3CA H1047R mutations by TIDE-Cas14a system detection. The fluorescence image shows the naked-eye result under blue light transilluminator. Data represent the means ± s.d. (n = 3). P values are determined by twotailed Student’s t-tests. ns., no significance; *P-value< 0.05, **P-value< 0.01, ***P-value< 0.001, ****P-value< 0.0001. Source data are provided as a Source Data file.


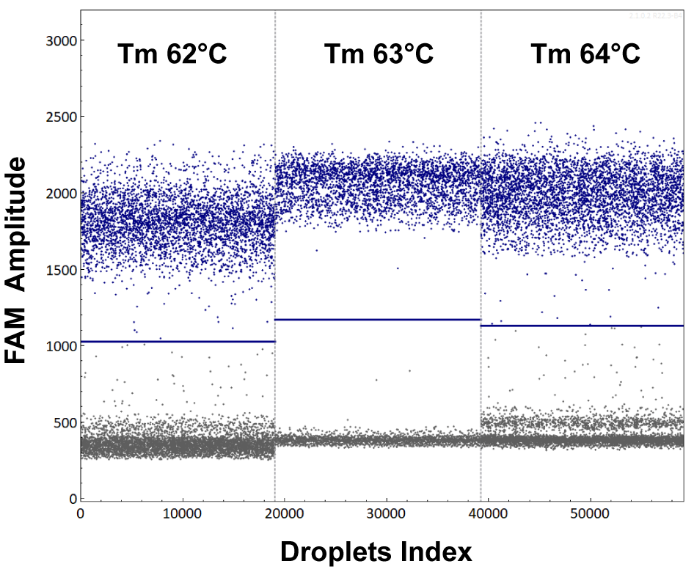


**Figure S7.** Performance analysis of TIDE-Cas14a system for the detection of PIK3CA H1047R mutation. Representative droplet plots demonstrating amplification efficiency and specificity of ddPCR primers across annealing temperatures (61-63°C). Source data are provided as a Source Data file.


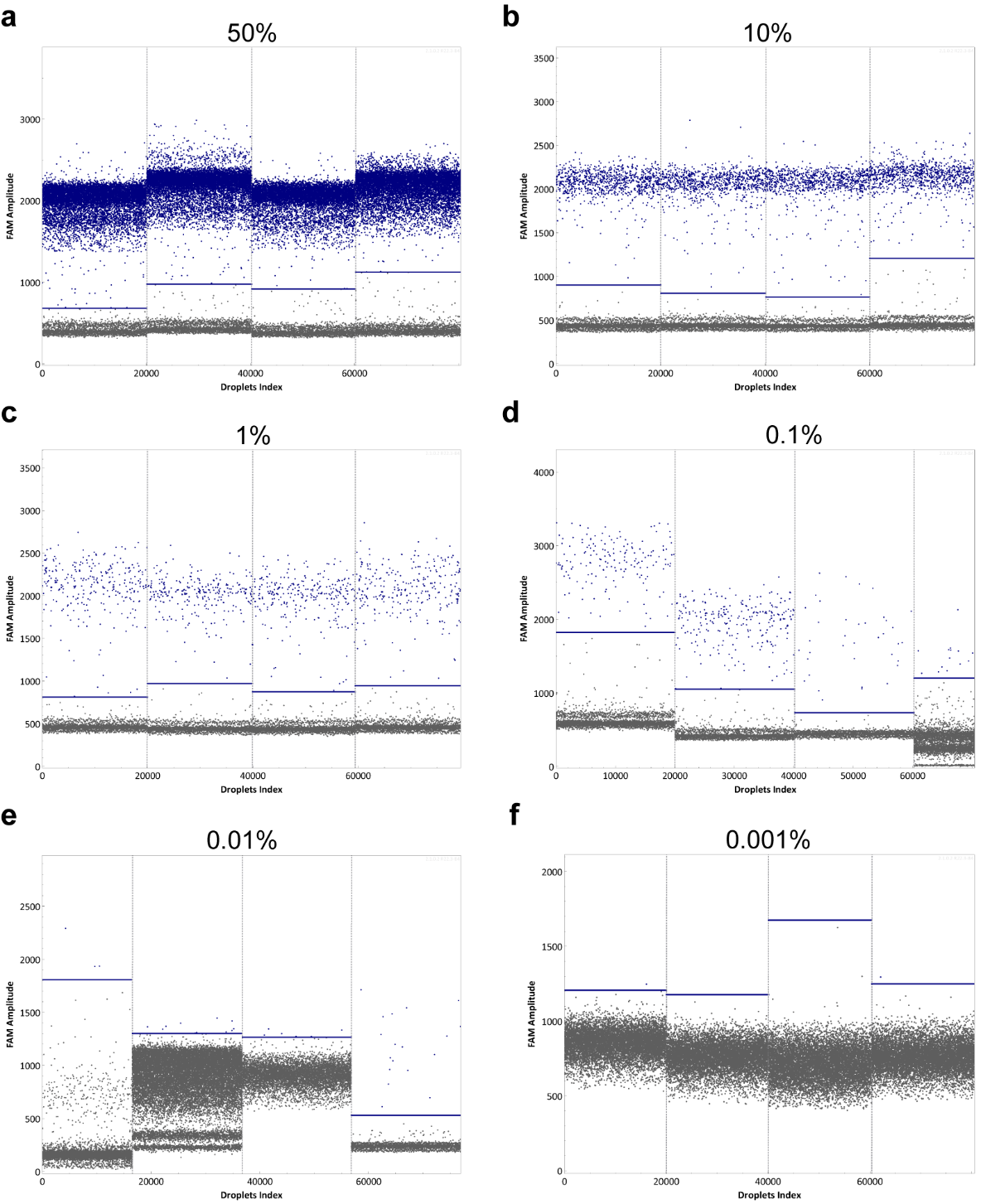


**Figure S8.** Optimization results of ddPCR LOD. LOD determination based on ddPCR analysis of DNA templates with VAFs ranging from 50% to 0.001% (n=4 replicates per VAF level).


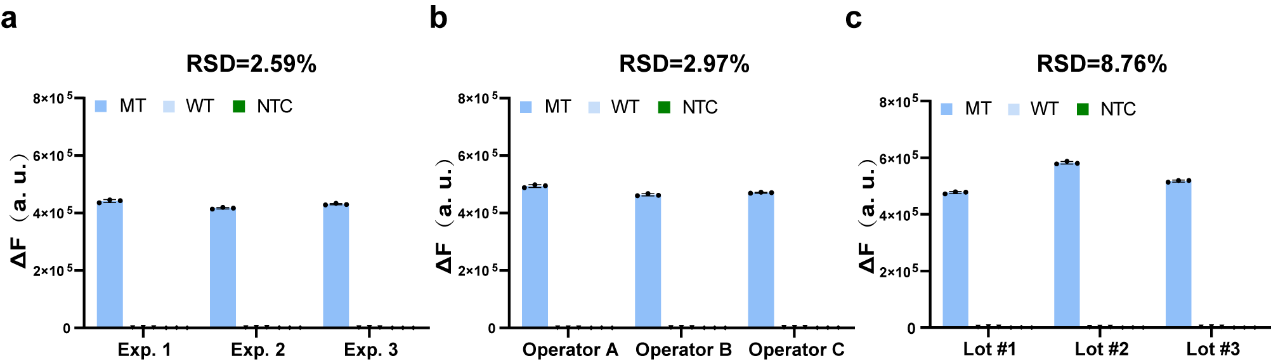


**Figure S9.** Stability assessment of TIDE-Cas14a system for EGFR L858R assay under variable conditions​​. a) Inter-day reproducibility test​​. Fluorescence signals of EGFR L858R detection across three independent experimental dates (Exp. 1-3). Relative standard deviation (RSD) = 2.59%. b) Inter-operator consistency test​​. Performance comparison among three trained operators (A-C) using identical reagent lots. RSD = 2.97%. c) Inter-lot reagent stability test​​. Consistency evaluation across three Cas14a protein production batches (Lot #1-3). RSD = 8.76%. NTC, no-template control. WT, wild template. MT, mutant template. Data represent the means ±s.d. (n = 3). Source data are provided as a Source Data file.


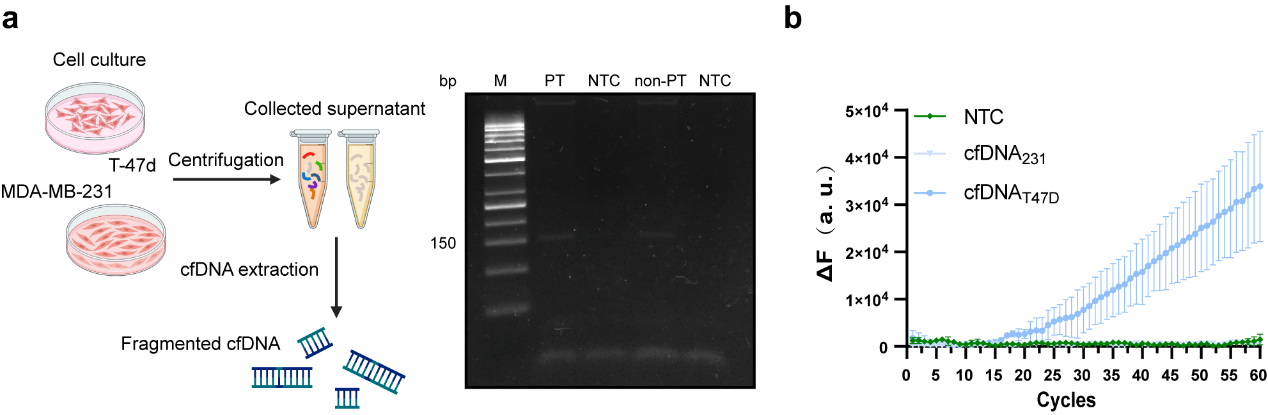


**Figure S10.** Selective detection of PIKK3CA H1047R in cfDNA samples using TIDE-Cas14a.**​​** a)​​ Enrichment efficiency comparison between PT-modified primers and conventional primers for cfDNA extracted from culture supernatants of T47D and MDA-MB-231 cells. Created in <https://BioRender.com> ​​b)​​ Signal intensity differences between T47D mutant cfDNA and MDA-MB-231 wild-type cfDNA. NTC, no-template control. Data in (b) represent the means ±s.d. (n = 3). Source data are provided as a Source Data file.


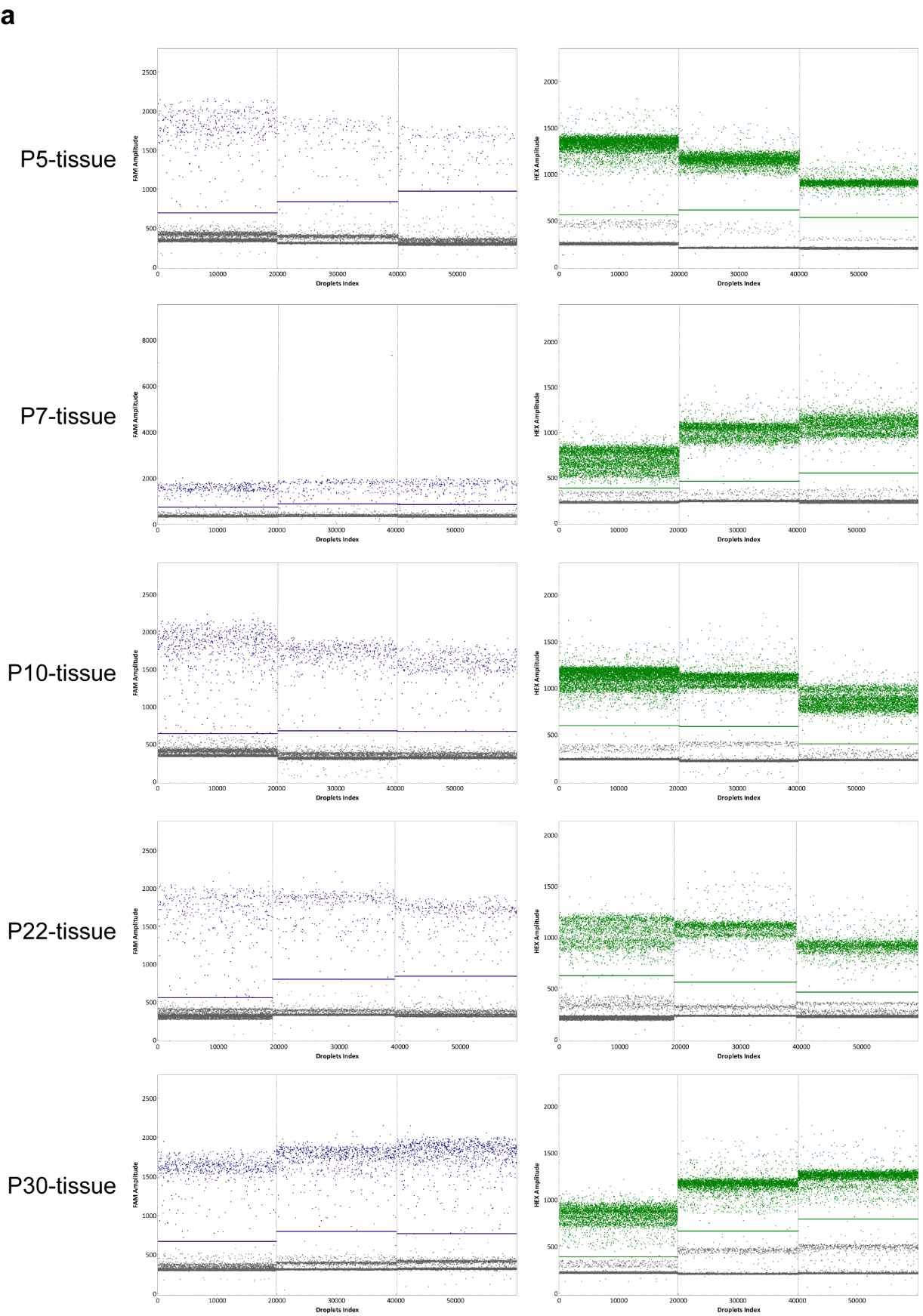


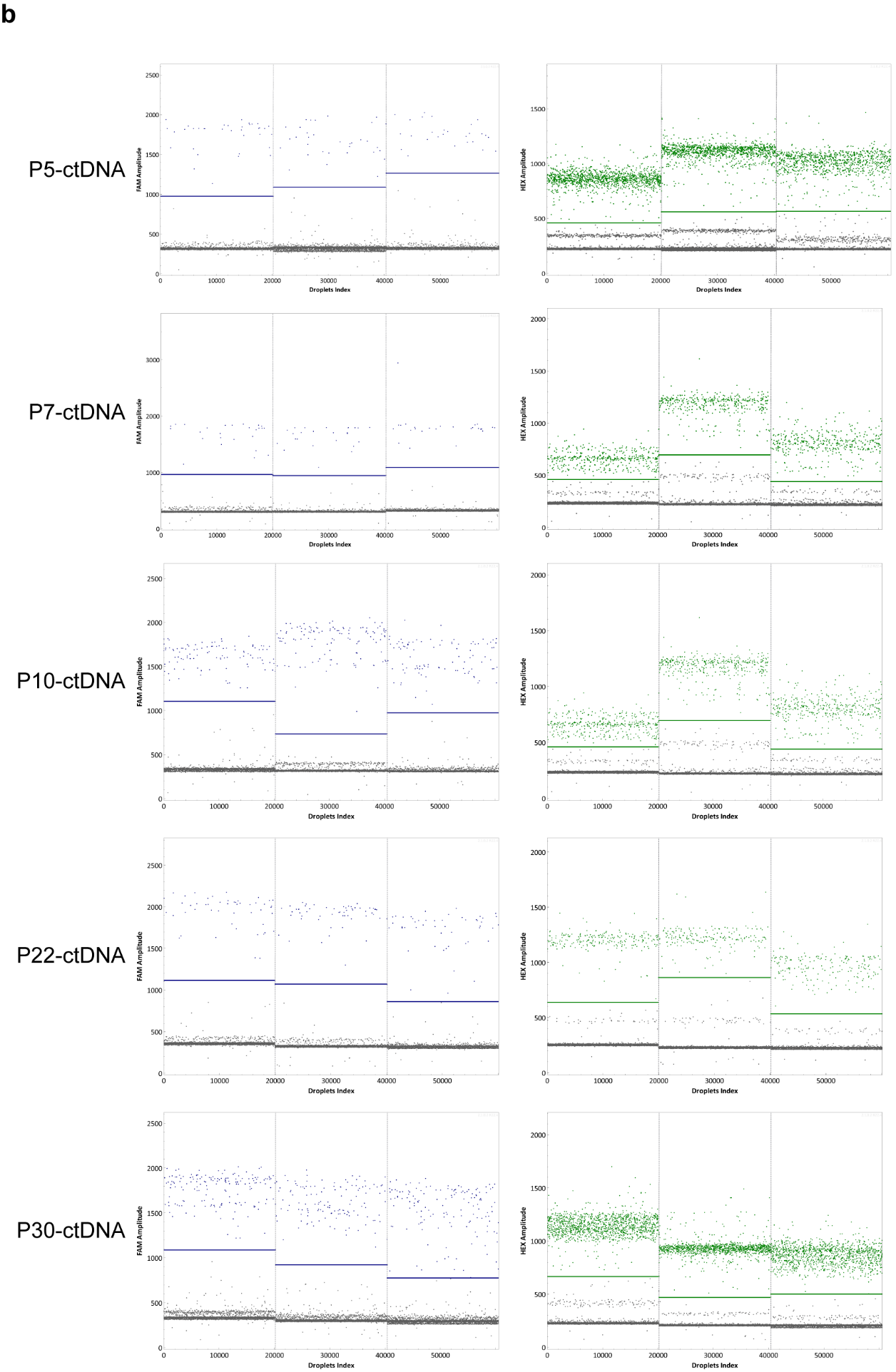


**Figure S11.** Detection of PIK3CA H1047R mutation in clinical samples using the ddPCR. a) 2D-plot results of positive clinical samples (P5, P7, P10, P22, P30) in tissue samples using the ddPCR. e) 2D-plot results of positive clinical samples (P5, P7, P10, P22, P30) in plasma samples using the ddPCR.


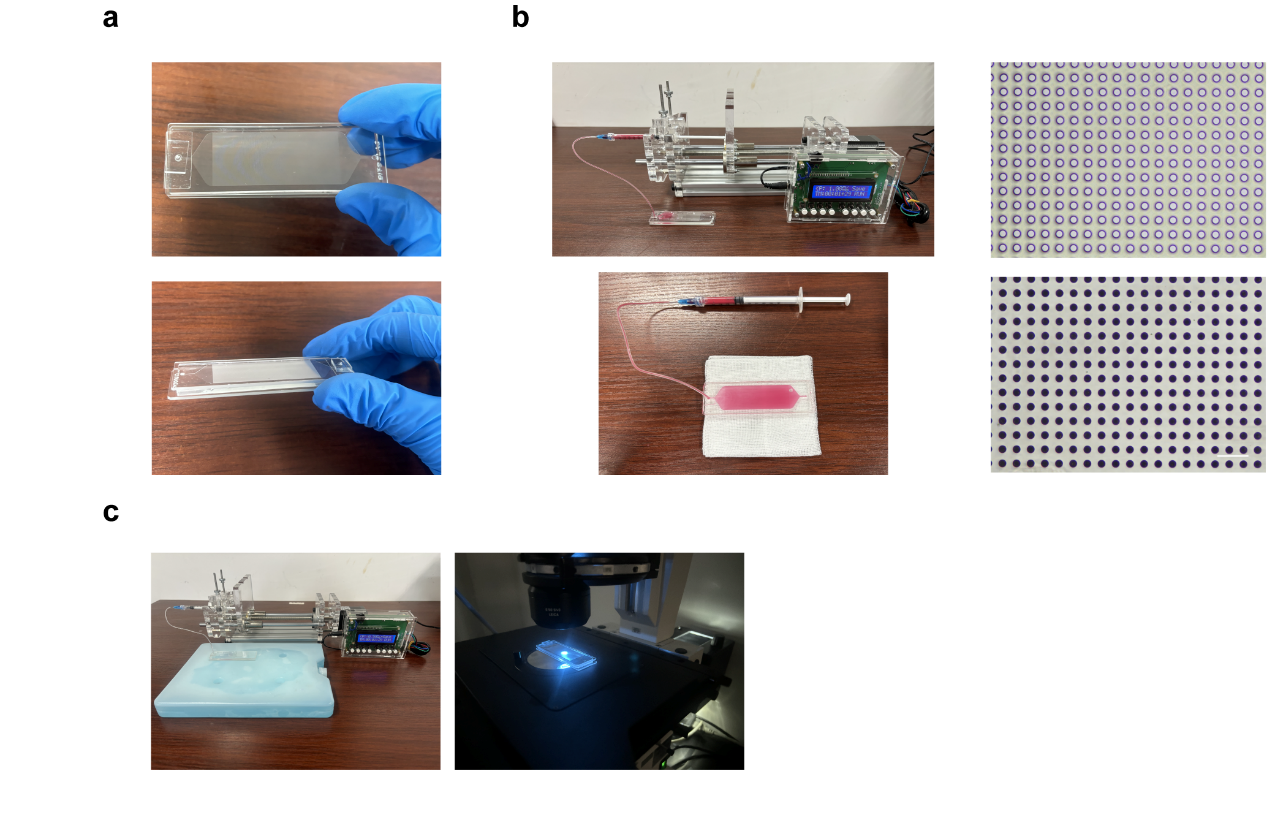


**Figure S12.** Schematics for the entire operation process for chip. a) Characterization of the Chip. b) Right: Loading of water-soluble dye; Left: Microscopy images for verifying filling efficiency of chip. c) On-ice loading of RPA-T7-CRISPR/Cas14a reagents and post-reaction chip observation. Scale bars are 100 μm.

**3. Supplementary Tables**

**Table S1.** Sequences of DNA or RNA oligonucleotides used in this work.

| Classification | Description^a, b^ | Nucleic Acid Sequence (5'→ 3') ^c, d^ |
| --- | --- | --- |
| PIK3CA  RPA Primer | F1 | T*T*G*CATACATTCGAAAGACCCTAGCCTTAGATA |
|  | R1 | TCAGTTCAATGCATGCTGTTTAATTGTGTGGA |
|  | F2 | A*A*C*TGAGCAAGAGGCTTTGGAGTATTTCATG |
|  | R2 | GCTGTTTAATTGTGTGGAAGATCCAATCCAT |
|  | R3 | AGTTATCTTTTCAGTTCAATGCATGCTGTTT |
|  | R4 | TCAGTTATCTTTTCAGTTCAATGCATGCTGT |
|  | R5 | CATGCTGTTTAATTGTGTGGAAGATCCAATC |
|  | E545K/E542K-F^[4]^ | C*T*C*AAAGCAATTTCTACACGAGATCCTCTC |
|  | E545K/E542K-R^[4]^ | AGCACTTACCTGTGACTCCATAGAAAATCT |
| EGFR T790M  RPA Primer^[5]^ | F1 | C*A*C*TGACGTGCCTCTCCCTCCCTCCAGGAAGCC |
|  | R1 | CCCTGATTACCTTTGCGATCTGCACACACCAG |
|  | F2 | G*G*C*ATCTGCCTCACCTCCAC |
|  | R2 | CCAATATTGTCTTTGTGTTCCCGGAC |
| EGFR L858R  RPA Primer^[5-9]^ | F1 | C*G*C*AGCATGTCAAGATCACAGATTTTGGGCG |
|  | R1 | ACCTAAAGCCACCTCCTTACTTTGCCTCCTTC |
|  | F2 | C*T*T*CTTCCCATGATGATCTGTCCCTCACAG |
|  | R2 | AAAGCCACCTCCTTACTTTGCCTCCTTCTG |
|  | F3 | T*G*A*AAACACCGCAGCATGTCAAGATCAC |
|  | R3 | ACCTAAAGCCACCTCCTTACTTTGCCTCC |
|  | F4 | C*A*G*AGCTTCTTCCCATGATGATCTGTCCCTC |
|  | R4 | CTGACCTAAAGCCACCTCCTTACTTTGCCTCC |
|  | F5 | C*C*T*CACAGCAGGGTCTTCTCTGTTTCAGGG |
|  | R5 | CTGGCTGACCTAAAGCCACCTCCTTACTTTGCC |
|  | F6 | A*A*C*ACCGCAGCATGTCAAGATCACAGATT |
|  | R6 | CCTCCTTCTGCATGGTATTCTTTCTCTTCC |
| BRAF V600E  RPA Primer^[10,11]^ | F1 | A*T*G*GGACCCACTCCATCGAG |
|  | R1 | ATAGGTGATTTTGGTCTAG |
|  | F2 | T*A*C*TGTTTTCCTTTACTTACTACACCTCAG |
|  | R2 | GACAACTGTTCAAACTGATGGGACCCACTC |
| KRAS G12V  RPA Primer^[12,13]^ | F1 | A*C*T*GGTGGAGTATTTGATAGTGTA |
|  | R1 | GTCCTGCACCAGTAATATGC |
|  | F2 | A*G*G*CCTGCTGAAAATGA |
|  | R2 | TGTTGGATCATATTCGTCCACA |
|  | F3 | G*G*T*GAGTTTGTATTAAAAGGTACTGG |
|  | R3 | TTAGCTGTATCGTCAAGGCACTC |
| PIK3CA H1047R  sgRNA Primer | sgRNA-F^e^ | TAATACGACTCACTATAGCTTCACTGATAAAGTGGAGAACCGCTTCACCAAAAGCTGTCCCTTAGGGGATTAGAACTTGAGTGAAGGTGGGCTGCTTGCATCAGCCTAATGTCGAGAAG |
|  | sgRNA-3-R | CAAATGAATGATGCAGGCCAGTTGCATTCCAAATGAATTTGTTTCGAGGGTTACTTTCCGAAGAAAGCACTTCTCGACATTAGGCTGAT |
|  | sgRNA-2-R | CAAATGAATGATGCAGGTGAGTTGCATTCCAAATGAATTTGTTTCGAGGGTTACTTTCCGAAGAAAGCACTTCTCGACATTAGGCTGAT |
|  | sgRNA-1-R | CAAATGAATGATGCAGGTCCGTTGCATTCCAAATGAATTTGTTTCGAGGGTTACTTTCCGAAGAAAGCACTTCTCGACATTAGGCTGAT |
|  | sgRNA-R | CAAATGAATGATGCACGTCAGTTGCATTCCAAATGAATTTGTTTCGAGGGTTACTTTCCGAAGAAAGCACTTCTCGACATTAGGCTGAT |
|  | sgRNA+1-R | CAAATGAATGATGCAGGTCAGTTGCATTCCAAATGAATTTGTTTCGAGGGTTACTTTCCGAAGAAAGCACTTCTCGACATTAGGCTGAT |
|  | sgRNA+2-R | CAAATGAATGATGCCCGTCAGTTGCATTCCAAATGAATTTGTTTCGAGGGTTACTTTCCGAAGAAAGCACTTCTCGACATTAGGCTGAT |
|  | sgRNA+3-R | CAAATGAATGATGGACGTCAGTTGCATTCCAAATGAATTTGTTTCGAGGGTTACTTTCCGAAGAAAGCACTTCTCGACATTAGGCTGAT |
| EGFR T790M  sgRNA Primer | sgRNA-3-R | ACCGTGCAGCTCATCATGCCGTTGCATTCCAAATGAATTTGTTTCGAGGGTTACTTTCCGAAGAAAGCACTTCTCGACATTAGGCTGAT |
|  | sgRNA-2-R | ACCGTGCAGCTCATCATGGAGTTGCATTCCAAATGAATTTGTTTCGAGGGTTACTTTCCGAAGAAAGCACTTCTCGACATTAGGCTGAT |
|  | sgRNA-1-R | ACCGTGCAGCTCATCATCCAGTTGCATTCCAAATGAATTTGTTTCGAGGGTTACTTTCCGAAGAAAGCACTTCTCGACATTAGGCTGAT |
|  | sgRNA-R | ACCGTGCAGCTCATCATGCAGTTGCATTCCAAATGAATTTGTTTCGAGGGTTACTTTCCGAAGAAAGCACTTCTCGACATTAGGCTGAT |
|  | sgRNA+1-R | ACCGTGCAGCTCATCCTGCAGTTGCATTCCAAATGAATTTGTTTCGAGGGTTACTTTCCGAAGAAAGCACTTCTCGACATTAGGCTGAT |
|  | sgRNA+2-R | ACCGTGCAGCTCATGATGCAGTTGCATTCCAAATGAATTTGTTTCGAGGGTTACTTTCCGAAGAAAGCACTTCTCGACATTAGGCTGAT |
|  | sgRNA+3-R | ACCGTGCAGCTCACCATGCAGTTGCATTCCAAATGAATTTGTTTCGAGGGTTACTTTCCGAAGAAAGCACTTCTCGACATTAGGCTGAT |
| EGFR L858R  sgRNA Primer | sgRNA-3-R | ATCACAGATTTTGGGCGGGGGTTGCATTCCAAATGAATTTGTTTCGAGGGTTACTTTCCGAAGAAAGCACTTCTCGACATTAGGCTGAT |
|  | sgRNA-2-R | ATCACAGATTTTGGGCGGCCGTTGCATTCCAAATGAATTTGTTTCGAGGGTTACTTTCCGAAGAAAGCACTTCTCGACATTAGGCTGAT |
|  | sgRNA-1-R | ATCACAGATTTTGGGCGCGCGTTGCATTCCAAATGAATTTGTTTCGAGGGTTACTTTCCGAAGAAAGCACTTCTCGACATTAGGCTGAT |
|  | sgRNA-R | ATCACAGATTTTGGGCGGGCGTTGCATTCCAAATGAATTTGTTTCGAGGGTTACTTTCCGAAGAAAGCACTTCTCGACATTAGGCTGAT |
|  | sgRNA+1-R | ATCACAGATTTTGGGGGGGCGTTGCATTCCAAATGAATTTGTTTCGAGGGTTACTTTCCGAAGAAAGCACTTCTCGACATTAGGCTGAT |
|  | sgRNA+2-R | ATCACAGATTTTGGCCGGGCGTTGCATTCCAAATGAATTTGTTTCGAGGGTTACTTTCCGAAGAAAGCACTTCTCGACATTAGGCTGAT |
|  | sgRNA+3-R | ATCACAGATTTTGCGCGGGCGTTGCATTCCAAATGAATTTGTTTCGAGGGTTACTTTCCGAAGAAAGCACTTCTCGACATTAGGCTGAT |
| BRAF V600E  sgRNA Primer | sgRNA-3-R | CGAGATTTCTCTCTAGCTAGGTTGCATTCCAAATGAATTTGTTTCGAGGGTTACTTTCCGAAGAAAGCACTTCTCGACATTAGGCTGAT |
|  | sgRNA-2-R | CGAGATTTCTCCGTAGCTAGGTTGCATTCCAAATGAATTTGTTTCGAGGGTTACTTTCCGAAGAAAGCACTTCTCGACATTAGGCTGAT |
|  | sgRNA-1-R | CGAGATTTCTGTGTAGCTAGGTTGCATTCCAAATGAATTTGTTTCGAGGGTTACTTTCCGAAGAAAGCACTTCTCGACATTAGGCTGAT |
|  | sgRNA-R | CGAGATTTCTCTGTAGCTAGGTTGCATTCCAAATGAATTTGTTTCGAGGGTTACTTTCCGAAGAAAGCACTTCTCGACATTAGGCTGAT |
|  | sgRNA+1-R | CGAGATTTGTCTGTAGCTAGGTTGCATTCCAAATGAATTTGTTTCGAGGGTTACTTTCCGAAGAAAGCACTTCTCGACATTAGGCTGAT |
|  | sgRNA+2-R | CGAGATTCCTCTGTAGCTAGGTTGCATTCCAAATGAATTTGTTTCGAGGGTTACTTTCCGAAGAAAGCACTTCTCGACATTAGGCTGAT |
|  | sgRNA+3-R | CGAGATCTCTCTGTAGCTAGGTTGCATTCCAAATGAATTTGTTTCGAGGGTTACTTTCCGAAGAAAGCACTTCTCGACATTAGGCTGAT |
| KRAS G12V  sgRNA Primer | sgRNA-3-R | ACTCTTGCCTACGCCAACACGTTGCATTCCAAATGAATTTGTTTCGAGGGTTACTTTCCGAAGAAAGCACTTCTCGACATTAGGCTGAT |
|  | sgRNA-2-R | ACTCTTGCCTACGCCAACCGGTTGCATTCCAAATGAATTTGTTTCGAGGGTTACTTTCCGAAGAAAGCACTTCTCGACATTAGGCTGAT |
|  | sgRNA-1-R | ACTCTTGCCTACGCCAAGAGGTTGCATTCCAAATGAATTTGTTTCGAGGGTTACTTTCCGAAGAAAGCACTTCTCGACATTAGGCTGAT |
|  | sgRNA-R | ACTCTTGCCTACGCCAACAGGTTGCATTCCAAATGAATTTGTTTCGAGGGTTACTTTCCGAAGAAAGCACTTCTCGACATTAGGCTGAT |
|  | sgRNA+1-R | ACTCTTGCCTACGCCCACAGGTTGCATTCCAAATGAATTTGTTTCGAGGGTTACTTTCCGAAGAAAGCACTTCTCGACATTAGGCTGAT |
|  | sgRNA+2-R | ACTCTTGCCTACGCGAACAGGTTGCATTCCAAATGAATTTGTTTCGAGGGTTACTTTCCGAAGAAAGCACTTCTCGACATTAGGCTGAT |
|  | sgRNA+3-R | ACTCTTGCCTACGGCAACAGGTTGCATTCCAAATGAATTTGTTTCGAGGGTTACTTTCCGAAGAAAGCACTTCTCGACATTAGGCTGAT |
| PIK3CA H1047R  crRNA | crRNA-3 | GGACGUGCAUCAUUCAUUUG |
|  | crRNA-2 | UCACGUGCAUCAUUCAUUUG |
|  | crRNA-1 | UGGCGUGCAUCAUUCAUUUG |
|  | crRNA | UGACGUGCAUCAUUCAUUUG |
|  | crRNA+1 | UGACCUGCAUCAUUCAUUUG |
|  | crRNA+2 | UGACGGGCAUCAUUCAUUUG |
|  | crRNA+3 | UGACGUCCAUCAUUCAUUUG |
| EGFR T790M  crRNA | crRNA-3 | GGCAUGAUGAGCUGCACGGU |
|  | crRNA-2 | UCCAUGAUGAGCUGCACGGU |
|  | crRNA-1 | UGGAUGAUGAGCUGCACGGU |
|  | crRNA | UGCAUGAUGAGCUGCACGGU |
|  | crRNA+1 | UGCAGGAUGAGCUGCACGGU |
|  | crRNA+2 | UGCAUCAUGAGCUGCACGGU |
|  | crRNA+3 | UGCAUGGUGAGCUGCACGGU |
| EGFR L858R  crRNA | crRNA-3 | CCCCGCCCAAAAUCUGUGAU |
|  | crRNA-2 | GGCCGCCCAAAAUCUGUGAU |
|  | crRNA-1 | GCGCGCCCAAAAUCUGUGAU |
|  | crRNA | GCCCGCCCAAAAUCUGUGAU |
|  | crRNA+1 | GCCCCCCCAAAAUCUGUGAU |
|  | crRNA+2 | GCCCGGCCAAAAUCUGUGAU |
|  | crRNA+3 | GCCCGCGCAAAAUCUGUGAU |
| BRAF V600E  crRNA | crRNA-3 | CUAGCUAGAGAGAAAUCUCG |
|  | crRNA-2 | CUAGCUACGGAGAAAUCUCG |
|  | crRNA-1 | CUAGCUACACAGAAAUCUCG |
|  | crRNA | CUAGCUACAGAGAAAUCUCG |
|  | crRNA+1 | CUAGCUACAGACAAAUCUCG |
|  | crRNA+2 | CUAGCUACAGAGGAAUCUCG |
|  | crRNA+3 | CUAGCUACAGAGAGAUCUCG |
| KRAS G12V  crRNA | crRNA-3 | GUGUUGGCGUAGGCAAGAGU |
|  | crRNA-2 | CGGUUGGCGUAGGCAAGAGU |
|  | crRNA-1 | CUCUUGGCGUAGGCAAGAGU |
|  | crRNA | CUGUUGGCGUAGGCAAGAGU |
|  | crRNA+1 | CUGUGGGCGUAGGCAAGAGU |
|  | crRNA+2 | CUGUUCGCGUAGGCAAGAGU |
|  | crRNA+3 | CUGUUGCCGUAGGCAAGAGU |
| PIK3CA H1047R  ddPCR Primer | F | AGAGGCTTTGGAGTATTTCATG |
|  | R | TGTGTGGAAGATCCAATCCA |
|  | M-Probe | 5' FAM-ACCATGACGTGCATC-3' MGB |
|  | W-Probe | 5' HEX-ACCATGATGTGCATC-3' MGB |

Note. a. F, Forward primer; R, Reverse primer. b. M, Mutant; W, Wild. c. *, phosphorothioate (PT) modification. d. 5'FAM, 5'-FAM (Fluorescein) modification; 5'HEX, 5'-HEX (Fluorescein) modification; 3' MGB, minor Groove Binder, which stabilizes DNA duplexes by binding the minor groove, increasing probe Tm by ~10-15°C and enhancing SNP discrimination. e. The sgRNA-F primer sequence is employed for PIK3CA H1047R, EGFR T790M, EGFR L858R, BRAF V600E, and KRAS G12V mutations.

**Table S2.** Comparison with reported CRISPR/Cas based biosensors for other SNP detection.

| **Assay** | **Target**  **Amplification** | **Cas effector** | **Number of steps** | **Assay time**  **(min)** | **Digital**  **quantification** | **Limit of detection (VAF)** | **SNV Clinical**  **validation** |
| --- | --- | --- | --- | --- | --- | --- | --- |
| TIDE-Cas14a | RPA | Cas14a | 1 | 60 | Yes | 0.01% | Yes |
| CASMART^[9]^ | RPA | Cas12a | 1 | 60 | Yes | 0.1% | Yes |
| SHERLOCK^[14]^ | RPA | Cas13a | 2 | 120 | No | 0.1% | No |
| CDetection^[15]^ | RPA | Cas12b | 2 | 60-80 | No | 1% | No |
| HOLMESv2^[16]^ | LAMP | Cas12b | 2 | ＜60 | No | NA | No |
| EasyCatch^[17]^ | RPA | Cas12a | 2 | ＜60 | No | 0.001% | Yes |
| C-SDA-CRISPR/Cas12a^[18]^ | C-SDA | Cas12a | 2 | 140 | No | 0.001% | No |
| Y. Liu, et al.^[19]^ | RPA | Cas12a | 2 | 40 | No | 0.1% | Yes |
| MAV-chip based CRISPR/Cas12a^[20]^ | No | Cas12a | 1 | 60 | No | 0.01% | No |

**Table S3.** 32 breast cancer patients’ information.

| Sample ID | Gender | Age | Clinical stage | Tumor size  (cm, greastest dimension) | ER | PR | HER-2 | Ki-67(%) | VAF^a^ (%) | | |
| --- | --- | --- | --- | --- | --- | --- | --- | --- | --- | --- | --- |
| 1 | Female | 54 | IIA | 1.5 | (+) | (-) | 3 | 40 | 1.39 | 1.10 | 1.04 |
| 2 | Female | 57 | IIB | 4 | (-) | (-) | 3 | 50 | 0.77 | 0.72 | 0.79 |
| 3 | Female | 42 | IIA | 1.5 | (+) | (+) | 1 | 20 | 0.97 | 0.87 | 0.81 |
| 4 | Female | 53 | IIA | 2 | (-) | (-) | 3 | 50 | 1.13 | 0.85 | 0.79 |
| 5 | Female | 44 | IA | 2 | (+) | (+) | 1 | 10 | 3.39 | 2.14 | 2.64 |
| 6 | Female | 44 | IIB | 2.5 | (+) | (+) | 1 | 30 | 0.98 | 1.38 | 1.14 |
| 7 | Female | 58 | IA | 1.5 | (+) | (+) | 2 | 5 | 2.80 | 2.68 | 4.10 |
| 8 | Female | 60 | IIB | 3 | (+) | (+) | 2 | 30 | 1.65 | 1.61 | 1.56 |
| 9 | Female | 47 | IIB | 2 | (+) | (+) | 1 | 25 | 2.67 | 2.47 | 2.36 |
| 10 | Female | 41 | IIA | 3 | (+) | (+) | 1 | 10 | 19.86 | 23.11 | 19.80 |
| 11 | Female | 53 | IIB | 1.5 | (-) | (-) | 2 | 20 | 1.94 | 1.42 | 1.91 |
| 12 | Female | 54 | IIA | 1.8 | (+) | (+) | 0 | 25 | 1.69 | 0.96 | 1.02 |
| 13 | Female | 77 | IA | 2 | (+) | (-) | 3 | 20 | 2.39 | 1.77 | 1.68 |
| 14 | Female | 77 | IA | 1.8 | (+) | (+) | 1 | 40 | 3.05 | 2.74 | 2.61 |
| 15 | Female | 55 | IIA | 3.5 | (-) | (-) | 3 | 10 | 2.36 | 1.93 | 1.68 |
| 16 | Female | 49 | IIA | 2.8 | (+) | (+) | 1 | 15 | 1.32 | 1.09 | 1.31 |
| 17 | Female | 72 | IIA | 2.4 | (+) | (+) | 1 | 20 | 3.12 | 2.52 | 3.32 |
| 18 | Female | 43 | IIA | 2.5 | (+) | (+) | 2 | 50 | 1.50 | 1.56 | 1.50 |
| 19 | Female | 50 | IA | 1.5 | (+) | (+) | 2 | 10 | 0.87 | 0.49 | 0.42 |
| 20 | Female | 69 | IIA | 1.8 | (+) | (+) | 1 | 30 | 3.62 | 2.24 | 2.27 |
| 21 | Female | 70 | IA | 2 | (-) | (-) | 1 | 30 | 0.96 | 0.97 | 0.89 |
| 22 | Female | 41 | IIA | 1 | (-) | (-) | 3 | 50 | 18.04 | 23.29 | 20.00 |
| 23 | Female | 53 | IIB | 1.5 | (-) | (-) | 2 | 20 | 0.51 | 0.68 | 0.89 |
| 24 | Female | 63 | IIB | 2.3 | (+) | (+) | 1 | 20 | 1.18 | 1.19 | 1.05 |
| 25 | Female | 57 | IIB | 4 | (-) | (-) | 3 | 50 | 1.38 | 1.51 | 1.24 |
| 26 | Female | 71 | IA | 1.8 | (+) | (+) | 1 | 10 | 1.09 | 0.41 | 1.07 |
| 27 | Female | 69 | IA | 2 | (+) | (+) | 1 | 15 | 0.36 | 2.06 | 2.38 |
| 28 | Female | 58 | IIA | 1.9 | (+) | (+) | 0 | 10 | 0 | 1.16 | 0 |
| 29 | Female | 52 | IIA | 2.1 | (+) | (+) | 0 | 10 | 0.47 | 0.21 | 0.55 |
| 30 | Female | 58 | IIA | 1.9 | (+) | (+) | 0 | 10 | 10.66 | 9.22 | 10.35 |
| 31 | Female | 48 | IIA | 2.2 | (+) | (+) | 0 | 40 | 0 | 0.67 | 2.36 |
| 32 | Female | 60 | IIA | 2.8 | (+) | (-) | 3 | 70 | 2.33 | 2.28 | 0.89 |

Note. a. VAF was determined by ddPCR from plasma.

**Table S4.** Stratified analysis of VAF in 32 plasma Samples by clinical variables.

| Clinical Variable | Subgroup | Sample Size | Mean VAF (%) | | |
| --- | --- | --- | --- | --- | --- |
| Tumor Stage | Stage IA | 8 | 1.43 | 1.32 | 1.47 |
|  | Stage IIA | 16 | 4.28 | 4.57 | 4.22 |
|  | Stage IIB | 8 | 1.82 | 1.71 | 1.87 |
| Tumor Size | ≤2 cm | 19 | 2.89 | 2.95 | 2.91 |
|  | >2 cm | 13 | 3.04 | 3.2 | 3.00 |
| ER Status | Positive | 24 | 2.67 | 2.64 | 2.59 |
|  | Negative | 8 | 3.82 | 4.26 | 4.03 |

**Table S5.** Various costs for a single test.

| **RPA** | **Price per kit** | **Volume/Reaction per kit** | **Volume per reaction** | **Cost per reaction** |
| --- | --- | --- | --- | --- |
| TwistAmp® Basic | $599 | 96 reactions | 1.0 reaction | $6.2395 |
| Primer F | $9.03 | 1000 μL | 2.4 μL | $0.0216 |
| Primer R | $7.75 | 1000 μL | 2.4 μL | $0.0185 |
| T7 exonuclease | $111.24 | 100μL (1000U) | 2.5 U/μL | $0.2781 |
| **CRISPR/Cas14a** | **Price per kit** | **Volume/Reaction per kit** | **Volume per reaction** | **Cost per reaction** |
| Cas14a | $557.58 | 100 μL | 1.25 μL | $6.9697 |
| sgRNA | $111.52 | 1000 μL | 1.25 μL | $0.1394 |
| ssDNA-FQ | $94.79 | 1200 μL | 1.25 μL | $0.0987 |
| **Chip** | **Price per chip** | **Volume of TIDE-Cas14a per chip** | **Size of per chip** | **Cost per reaction** |
|  | $2.79 | 100ul | 74mm*25mm*4mm | $43.7091 |
|  |  |  | Total: | $46.4991 |

**4. References**

[1] I. Keraite, V. Alvarez-Garcia, I. Garcia-Murillas, M. Beaney, N. C. Turner, C. Bartos, O. Oikonomidou, M. Kersaudy-Kerhoas, N. R. Leslie, PIK3CA mutation enrichment and quantitation from blood and tissue, *Sci Rep* **2020**, *10, 17082*.

[2] Y. Yan, D. Zhang, P. Zhou, B. Li, S. Y. Huang, HDOCK: a web server for protein-protein and protein-DNA/RNA docking based on a hybrid strategy, *Nucleic Acids Res* **2017**, *45, W365-w373*.

[3] Y. Yan, H. Tao, J. He, S. Y. Huang, The HDOCK server for integrated protein-protein docking, *Nat Protoc* **2020**, *15, 1829-1852*.

[4] V. Thoeny, E. Melnik, M. Huetter, M. Asadi, P. Mehrabi, T. Schalkhammer, W. Pulverer, T. Maier, G. C. Mutinati, P. Lieberzeit, R. Hainberger, Recombinase polymerase amplification in combination with electrochemical readout for sensitive and specific detection of PIK3CA point mutations, *Anal Chim Acta* **2023**, *1281, 341922*.

[5] Y. Ma, Y. Chu, Z. Xu, C. Xie, X. Ma, L. Zhang, J. Hu, B. Zou, H. Wu, G. Zhou, Ultrafast and Highly Specific Detection of One-Base Mutated Cell-Free DNA at a Very Low Abundance, *Anal Chem* **2024**, *96, 117-126*.

[6] J. Y. Lee, B. H. Jeong, H. S. Jung, T. Kang, Y. Park, J. K. Rho, S. G. Park, M. Y. Lee, Highly Sensitive 3D-Nanoplasmonic-Based Epidermal Growth Factor Receptor Mutation Multiplex Assay Chip for Liquid Biopsy, *Small Sci* **2024**, *4, 2400101*.

[7] J. He, X. Hu, X. Weng, H. Wang, J. Yu, T. Jiang, L. Zou, X. Zhou, Z. Lyu, J. Liu, P. Zhou, X. Xiao, D. Zhen, Z. Deng, Efficient, specific and direct detection of double-stranded DNA targets using Cas12f1 nucleases and engineered guide RNAs, *Biosens Bioelectron* **2024**, *260, 116428*.

[8] Y. Tan, D. Huang, G. A. Wang, C. Shen, H. Deng, F. Li, Concentration-Bias-Free Discrimination of Single Nucleotide Variants Using Isothermal Nucleic Acid Amplification and Mismatch-Guided DNA Assembly, *Anal Chem* **2025**, *97, 1917-1924*.

[9] C. Zhang, Z. Cai, Z. Zhou, M. Li, W. Hong, W. Zhou, D. Yu, P. Wei, J. He, Y. Wang, C. Huang, X. Wang, J. Wu, CASMART, a one-step CRISPR Cas12a-mediated isothermal amplification for rapid and high-resolution digital detection of rare mutant alleles, *Biosens Bioelectron* **2023**, *222, 114956*.

[10] L. Zhang, J. Peng, J. Chen, L. Xu, Y. Zhang, Y. Li, J. Zhao, L. Xiang, Y. Ge, W. Cheng, Highly Sensitive Detection of Low-Abundance BRAF V600E Mutation in Fine-Needle Aspiration Samples by Zip Recombinase Polymerase Amplification, *Anal Chem* **2021**, *93, 5621-5628*.

[11] A. Etemadzadeh, P. Salehipour, F. M. Motlagh, M. Khalifeh, A. Asadbeigi, M. Tabrizi, R. Shirkouhi, M. H. Modarressi, An Optimized CRISPR/Cas12a Assay to Facilitate the BRAF V600E Mutation Detection, *J Clin Lab Anal* **2024**, *38, e25101*.

[12] J. Chen, T. Qiud, M. G. Mauk, Z. Su, Y. Fan, D. J. Yuan, Q. Zhou, Y. Qiao, H. H. Bau, J. Ying, J. Song, Programmable endonuclease combined with isothermal polymerase amplification to selectively enrich for rare mutant allele fractions, *Chin Chem Lett* **2022**, *33, 4126-4132*.

[13] R. Sebuyoya, S. Sevcikova, B. Yusuf, M. Bartosik, Integrating isothermal amplification techniques and LNA-based AI-assisted electrochemical bioassay for analysis of KRAS G12V point mutation, *Talanta* **2025**, *288, 127709*.

[14] J. S. Gootenberg, O. O. Abudayyeh, J. W. Lee, P. Essletzbichler, A. J. Dy, J. Joung, V. Verdine, N. Donghia, N. M. Daringer, C. A. Freije, C. Myhrvold, R. P. Bhattacharyya, J. Livny, A. Regev, E. V. Koonin, D. T. Hung, P. C. Sabeti, J. J. Collins, F. Zhang, Nucleic acid detection with CRISPR-Cas13a/C2c2, *Science* **2017**, *356, 438-442*.

[15] F. Teng, L. Guo, T. Cui, X. G. Wang, K. Xu, Q. Gao, Q. Zhou, W. Li, CDetection: CRISPR-Cas12b-based DNA detection with sub-attomolar sensitivity and single-base specificity, *Genome Biol* **2019**, *20, 132*.

[16] L. Li, S. Li, N. Wu, J. Wu, G. Wang, G. Zhao, J. Wang, HOLMESv2: A CRISPR-Cas12b-Assisted Platform for Nucleic Acid Detection and DNA Methylation Quantitation, *ACS Synth Biol* **2019**, *8, 2228-2237*.

[17] Y. Liu, Y. Chen, L. Dang, Y. Liu, S. Huang, S. Wu, P. Ma, H. Jiang, Y. Li, Y. Pan, Y. Wei, X. Ma, M. Liu, Q. Ji, T. Chi, X. Huang, X. Wang, F. Zhou, EasyCatch, a convenient, sensitive and specific CRISPR detection system for cancer gene mutations, *Mol Cancer* **2021**, *20, 157*.

[18] Y. Deng, G. Cao, X. Chen, M. Yang, D. Huo, C. Hou, Ultrasensitive detection of gene-PIK3CA(H1047R) mutation based on cascaded strand displacement amplification and trans-cleavage ability of CRISPR/Cas12a, *Talanta* **2021**, *232, 122415*.

[19] Y. Liu, Y. Chen, S. Huang, X. Ma, X. Huang, X. Wang, F. Zhou, Rapid and Sensitive Diagnosis of Drug-Resistant FLT3-F691L Mutation by CRISPR Detection, *Front Mol Biosci* **2021**, *8, 753276*.

[20] N. Shao, X. Han, Y. Song, P. Zhang, L. Qin, CRISPR-Cas12a Coupled with Platinum Nanoreporter for Visual Quantification of SNVs on a Volumetric Bar-Chart Chip, *Anal Chem* **2019**, *91, 12384-12391*.
